# Asymptomatic infections with *Chlamydia trachomatis*, *Neisseria gonorrhoeae*, and *Trichomonas vaginalis* among women in low- and middle-income countries: A systematic review and meta-analysis

**DOI:** 10.1371/journal.pgph.0003226

**Published:** 2024-05-23

**Authors:** Camille Fortas, Elisabeth Delarocque-Astagneau, Rindra Vatosoa Randremanana, Tania Crucitti, Bich-Tram Huynh

**Affiliations:** 1 Epidemiology and Modelling of Antibiotic Evasion Unit (EMAE), Institut Pasteur, Université Paris Cité, Paris, France; 2 Anti-infective Evasion and Pharmacoepidemiology Team, UVSQ, Inserm, CESP, Université Paris-Saclay, Montigny-le-Bretonneux, France; 3 University Department of Public Health, Prevention, Observation, Territories—UFR Simone Veil—Santé, Université Versailles Saint-Quentin-en-Yvelines, Montigny-le-Bretonneux, France; 4 Département Hospitalier D’épidémiologie et de Santé Publique, Hôpital Raymond-Poincaré, Groupe Hospitalier Universitaire Université Paris-Saclay, Assistance Publique- Hôpitaux de Paris, Garches, France; 5 Unit of Epidemiology and Clinical Research, Institut Pasteur de Madagascar, Antananarivo, Madagascar; 6 Unit of Experimental Bacteriology, Institut Pasteur de Madagascar, Antananarivo, Madagascar; Asian University for Women, BANGLADESH

## Abstract

Syndromic management of sexually transmitted infections (STIs) is common in settings with limited access to diagnostic testing. However, this approach does not capture asymptomatic STIs. Untreated asymptomatic infections may result in serious complications and sequelae in women. We aimed to estimate the proportion and the prevalence of asymptomatic *Chlamydia trachomatis* (CT), *Neisseria gonorrhoeae* (NG), and *Trichomonas vaginalis* (TV) infections among women in low- and middle-income countries. We searched Medline, Scopus, and Web of Science for articles published between 2000 and 2022. We used random effect models to compute the proportion and prevalence estimates and performed sub-group analysis. We evaluated the quality of each article using the Appraisal tool for Cross-Sectional Studies and performed sensitivity analyses. This study was registered with PROSPERO, CRD42022286673. Forty-eight eligible studies were included. The proportion of asymptomatic CT, NG, and TV infections were: 60.7% [95% Confidence Interval (CI): 50.4; 70.5], 53.3% [37.1; 69.1], and 56.9% [44.6; 68.9], respectively. The proportion of women with asymptomatic infections was the highest in Africa for the three pathogens. The pooled prevalence of asymptomatic CT, NG, and TV infection was 4.70 per 100 women [95%CI: 3.39; 6.20], 3.11 [1.34; 5.54], and 5.98 [3.46; 9.12], respectively. More than half of the women infected by CT, NG, or TV were asymptomatic. To avoid undiagnosed and untreated asymptomatic infections leading to complications, alternative approaches to syndromic management urgently need to be considered.

## Introduction

More than one million sexually transmitted infections (STI) are acquired everyday worldwide [1]. Most of the infections by *Chlamydia trachomatis* (CT), *Neisseria gonorrhoeae* (NG), and *Trichomonas vaginalis* (TV) occur in low- and middle-income countries (LMICs) [2].

The gold standard to detect these STIs are the Nucleic Acid Amplification Tests (NAATs); other methods include microscopy and bacterial culture [3]. Whilst high-income countries have developed screening programs [4] and use molecular methods, in most LMICs access to biological methods is hindered by the cost of diagnostics, lack of laboratory facilities and infrastructure, and trained personnel [5].

Therefore, STI detection and treatment in LMICs often rely on the syndromic management [6, 7].However, asymptomatic STI cases are not captured by this approach, leaving a fraction of those infected undiagnosed and untreated.

In women, untreated infections caused by CT and NG can result in serious complications such as pelvic inflammatory disease (PID), infertility, and ectopic pregnancy [8]. Untreated trichomoniasis in pregnant women is associated with preterm birth or low birth weight [9]. These STIs are also associated with a higher risk of acquiring and transmitting other STIs, including HIV [10]. Lastly, untreated infections sustain the transmission.

To date, no study has provided pooled estimates of the burden of asymptomatic infections of the three most common curable STIs.

In this perspective, we conducted a systematic review and a meta-analysis to estimate: (i) the proportion of asymptomatic women among those infected by CT, NG, and TV and, (ii) the prevalence of asymptomatic CT, NG, and TV infections in the female population in LMICs.

## Methods

### Search strategy

We searched MEDLINE, Web of Science, and Scopus databases. Search terms combined pathogen and disease terms, terms related to the absence of symptoms and countries ever classified as LMICs by the World Bank between 2000 and 2022 [11] (full search terms in S1 Text). We also screened references for eligible articles. When the estimates were collected but not presented, we contacted the authors up to three times.

### Definitions

We defined “proportion of asymptomatic infection” as the number of women who tested positive for a pathogen and were asymptomatic divided by the number of women positive for that pathogen. We defined “prevalence of asymptomatic infection” as the number of women who tested positive for a pathogen and were asymptomatic divided by the number of women tested for that pathogen (S2 Text).

We defined symptoms as manifestations of a disease that were reported by the participant, while signs were considered features of a disease observed by a healthcare provider.

### Selection criteria

We selected studies published between 1 January 2000 and 31 December 2022, in English and French. We included studies which tested for STIs using genital or urine specimens. We kept studies that used NAATs for the diagnosis of CT, NG, and TV [3]. For TV, we also included articles that used InPouch or performed wet mount microscopy immediately after collection, as these techniques showed fair sensitivity and specificity [12] and remain common detection methods in LMICs [13].

We selected studies where the investigator actively asked about at least one symptom, or where participants self-reported symptoms. We excluded articles in which authors assumed the absence of symptoms in the entire study population without collecting such information. Finally, we excluded articles when “asymptomatic” was defined as the absence of clinical signs as reported by the healthcare provider rather than by the women as we intended a participant-centred approach.

BTH, TC, RR, and CF conducted initial screening of titles and abstracts in pairs and inclusion conflicts were resolved through consensus. CF performed data extraction, and BTH, TC, and RR ensured exactness and validity. When multiple publications on the same study population and outcomes were found, we prioritized the most comprehensive and recent data. Extracted data included the number of asymptomatic positive women, total women tested, and women positive for a specific STI. Other variables we extracted were: first author, publication year, study year, country, recruitment site, number and type of reported symptoms, definition of asymptomatic women, rural/urban setting, specific populations (female sex works (FSWs), adolescents, women with HIV, adolescents, infertile women), age range, specimen type, other STI pathogens tested for, and detection techniques.

### Data analysis

We described regions, study designs, settings, recruitment sites, specific populations, number of symptoms, sampling methods, and laboratory procedures of the selected studies.

Proportion and prevalence of asymptomatic CT, NG, and TV infections were estimated first for all studies and excluding studies on FSWs, women with HIV, and women attending an STI clinic as they are not representative of the general population and have a higher risk of having an STI. We referred to these groups as “populations with an increased risk of STIs”. Separate estimations were conducted for FSWs, women with HIV as well as other key populations such as pregnant women, adolescents, and infertile women.

Random-effect models were applied for each of the above analyses using the DerSimonian and Laird variance estimator. As some figures were near 0 or 1, the Freeman-Tukey double arcsine transformation was applied. Statistical heterogeneity was evaluated using the χ^2^ test with Cochran’s Q and I^2^. Analyses and forest plots were conducted using *meta* and *metafor* packages in R software (version 4.1.1). Proportions and prevalence maps were generated using qGIS (version 3.22).

Subgroup analyses were performed by region, country income level, rural/urban setting, study period, and the number of symptoms assessed in the study. The role of these study variables in the heterogeneity of the estimates were explored in meta-regressions.

The risk of bias was assessed using the appraisal tool for cross-sectional studies (AXIS) using >70% of validated items as a cut-off to qualify studies with a low risk of bias [14]. We conducted a sensitivity analysis to assess the impact of excluding studies with a high risk of bias on our overall estimates.

Publication bias was evaluated by Funnel plots and the Egger test.

This article was written following the PRISMA checklist (S1 Table).

## Results

**Fig 1:**
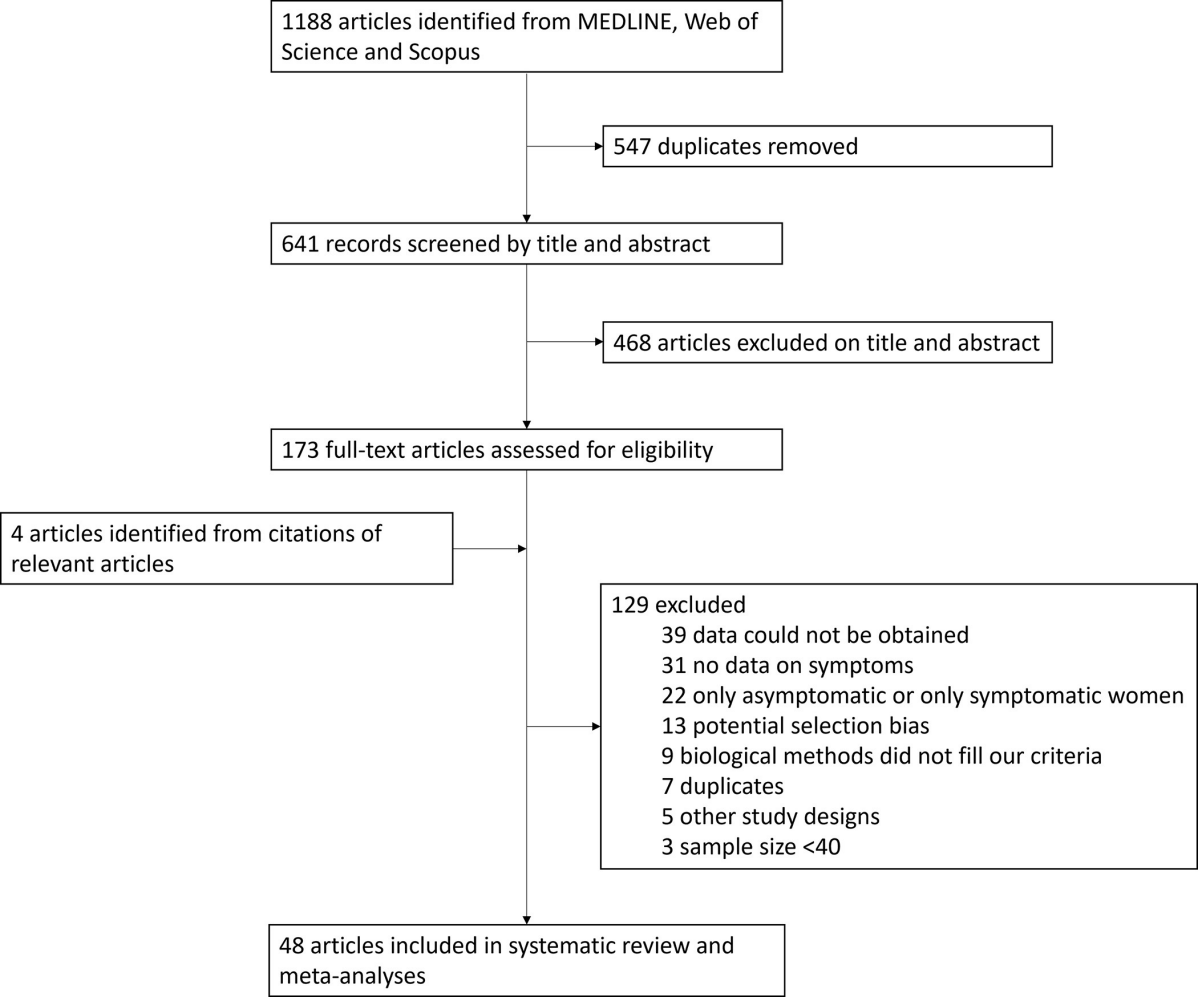
Study selection.

We identified 1188 articles, among which 547 were duplicates. We eliminated 468 articles based on title and abstract and screened 173 full texts and their citations. We found 4 additional relevant citations among selected articles. In total, 177 articles were eligible. Of those, 42 had available and extractable data and 45 reported having data according to our criteria but did not present the numbers. We were able to contact 40 of those 45 authors and retrieved data from six articles. In total, we used data from 48 studies corresponding to 99 data points for both the proportion and the prevalence: 41 for CT, 27 for NG, and 31 for TV (Fig 1, S2 Table, S3 Text). Twenty-one countries were represented in our study: eight in Africa, seven in Asia, five in Latin America, and one in Oceania. Most studies were cross-sectional (44/48, 92%) and few were cohort (2/48, 4%) or randomized controlled trials (2/48, 4%). Almost three-quarters of the studies (34/48, 71%) took place in urban settings, 21% (10/48) in rural settings, and 8% (4/48) in mixed settings. Hospitals and antenatal care were the most frequent recruitment sites (14/48, 29% and 11/48, 23%, respectively), followed by primary or secondary health care centres (9/48, 19%), locations where people with a high risk of STI can be found such as FSW venues, HIV centres or STI clinics (8/48, 17%). Six studies performed random sampling within the community (6/48, 13%). Some studies focused on specific populations: 15/48 (31%) were on pregnant women, 5/48 (10%) on FSWs, 4/48 (8%) on adolescents, 4/48 (8%) on women with HIV, and 4/48 (8%) on infertile women exclusively. Studies had enrolled between 48 and 4812 participants, and their age ranged from 10 to 90 years old although this information was unavailable for 14 studies.

The number of recorded symptoms ranged from one to 14, with more than half of the studies (23/48, 48%) exploring 1–4 symptoms.

Genital specimens (41/48, 85%) were the most common specimens, and few studies used urine (7/48, 15%). All techniques used for the laboratory diagnosis of CT (n = 41) and NG (n = 27) were NAATs, as per research criterium. For TV, NAATs were used in 25/31 studies, wet mount microscopy in 4/31, and InPouch in 2/31 studies.

**Fig 2:**
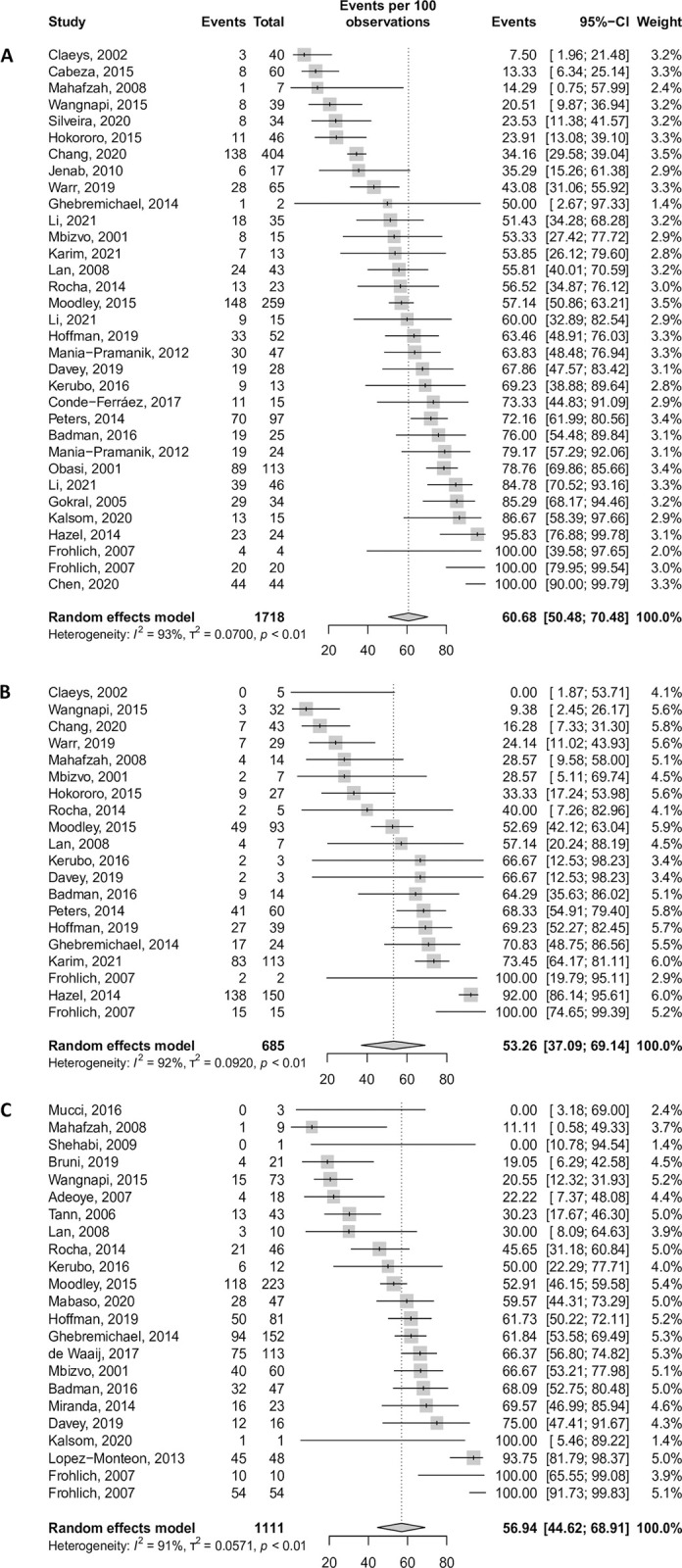
Forest plots of the proportion of asymptomatic CT (A), NG (B) and TV (C), in women, in LMICs.

**Fig 3:**
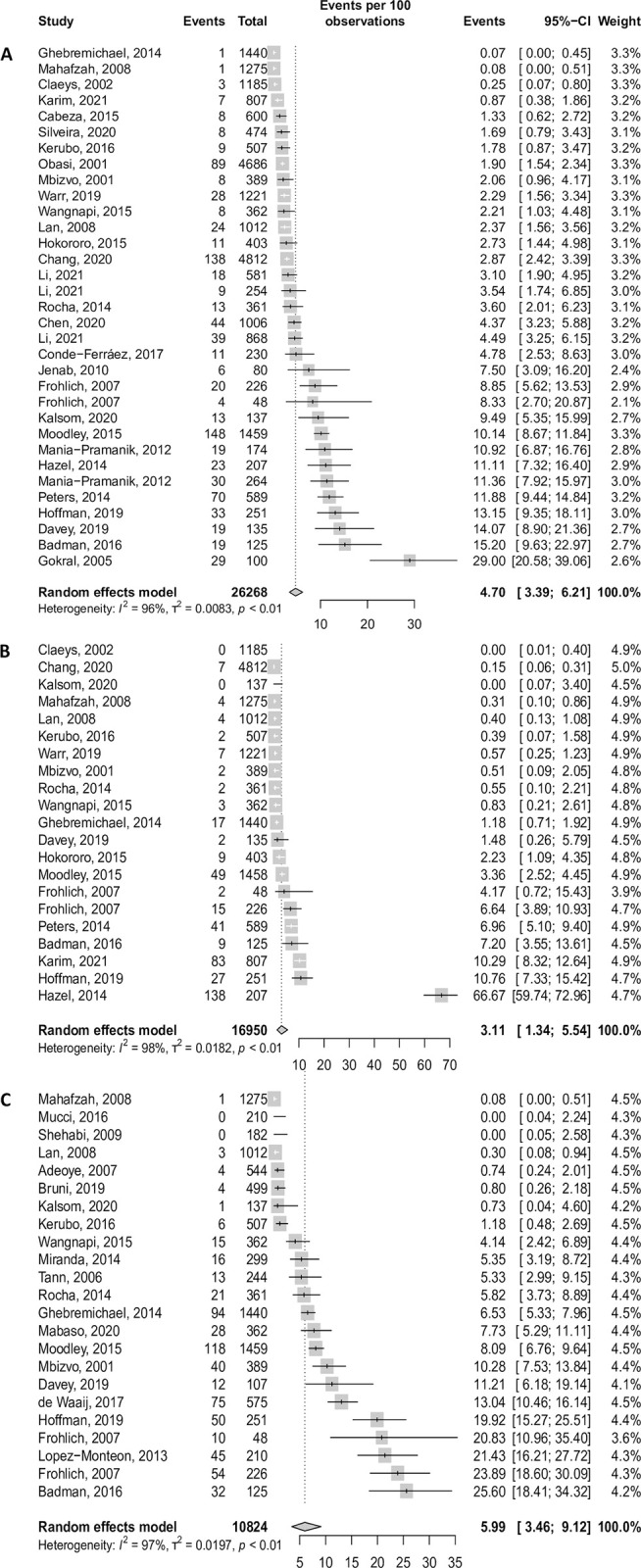
Forest plots of the prevalence of asymptomatic CT (A), NG (B) and TV (C), in women, in LMICs.

The estimates summarised below are presented in Table 1, Figs 2 and 3, and S3–S5 Tables. The proportion of asymptomatic CT, NG, and TV infections, excluding populations with an increased risk of STIs, was 60.7% 95%CI [50.4; 70.5] (33 data points, 26 268 participants), 53.3% [37.1; 69.1] (21 data points, 16 950 participants), and 56.9% [44.6; 68.9] (23 data points, 10 824 participants), respectively.

**Table 1:** Proportion and prevalence of asymptomatic CT, NG, and TV infections in women in LMICs.

		Proportion of asymptomatic infections			Prevalence of asymptomatic infections		
		(per 100 infected women) [95%CI]			(per 100 tested women) [95%CI]		
		CT	NG	TV	CT	NG	TV
**Overall**							
	Excluding populations with an increased risk of STI*	60.7 [50.4; 70.5]	53.3 [37.1; 69.1]	56.9 [44.6; 68.9]	4.70 [3.39; 6.20]	3.11 [1.34; 5.54]	5.98 [3.46; 9.12]
	Including populations with an increased risk of STI	58.8 [50.4; 67.0]	52.2 [38.5; 65.6]	53.6 [43.0; 64.1]	6.05 [4.26; 8.12]	3.70 [1.79; 6.23]	5.84 [3.75; 8.34]
Continent*							
	Africa	68.6 [56.4; 79.8]	67.2 [51.0; 81.9]	64.9 [52.2; 76.7]	4.99 [2.70; 7.90]	5.67 [2.15; 10.66]	9.06 [5.71; 13.07]
	Asia	66.2 [47.2; 83.0]	27.7 [9.0; 50.9]	17.3 [0.0; 49.5]	5.55 [3.40; 8.19]	0.13 [0.03; 0.28]	0.08 [0.00; 0.31]
	Latin America	30.9 [11.2; 54.8]	14.0 [0.0; 67.5]	50.1 [16.4; 83.8]	1.89 [0.56; 3.91]	0.12 [0.00; 1.16]	4.54 [0.52; 11.9]
	Oceania	47.4 [2.7; 95.0]	32.7 [0.0; 88.3]	43.3 [4.8; 87.5]	7.22 [0.00; 24.73]	3.12 [0.00; 12.25]	12.77 [0.06; 40.12]
Country income level*							
	Low-Income	55.5 [37.3; 73.1]	37.7 [18.4; 58.8]	45.4 [29.7; 61.6]	3.55 [1.87; 5.70]	0.57 [0.17; 1.17]	3.12 [0.73; 7.02]
	Middle-Income	63.2 [50.5; 75.1]	60.9 [41.1; 79.2]	61.7 [45.4; 77.1]	5.34 [3.59; 7.39]	5.21 [1.77; 10.22]	7.28 [3.72; 11.88]
Setting*							
	Rural	71.6 [55.6; 85.3]	70.4 [46.3; 90.3]	75.0 [49.8; 94.0]	5.36 [3.11; 8.15]	6.80 [1.39; 15.60]	10.27 [2.41; 22.46]
	Urban	55.6 [43.8; 67.1]	44.4 [26.5; 62.9]	58.9 [46.5; 70.8]	4.83 [2.90; 7.19]	1.33 [0.29; 3.02]	4.81 [2.25; 8.21]
Study year*							
	1998–2011	61.8 [48.1; 74.7]	55.9 [32.9; 77.8]	57.3 [41.0; 73.0]	4.41 [2.37; 6.99]	3.72 [0.98; 7.99]	5.94 [2.86; 10.00]
	2011–2022	57.7 [42.1; 72.7]	48.9 [26.9; 71.1]	61.1 [41.3; 79.5]	4.09 [2.88; 5.49]	2.42 [0.47; 5.63]	6.99 [2.09; 14.30]
Number of symptoms assessed*							
	Between 1 and 4	57.4 [41.6; 72.4]	40.6 [13.8; 70.4]	55.1 [37.7; 71.9]	6.53 [3.46; 10.45]	4.39 [0.46; 11.76]	5.68 [2.97; 9.17]
	Five and more	69.0 [51.7; 84.2]	71.8 [56.6; 85.2]	63.6 [43.3; 81.9]	4.29 [2.48; 6.53]	3.50 [1.27; 6.68]	7.66 [2.94; 14.24]
**Key population†**							
	Pregnant women	55.3 [40.6; 69.6]	45.7 [27.9; 63.9]	58.8 [43.9; 73.0]	7.50 [4.31; 11.44]	2.60 [1.27; 4.32]	7.96 [4.73; 11.91]
	Female sex workers	45.5 [24.8; 67.0]	38.1 [3.1; 82.9]	26.7 [19.6; 34.5]	13.63 [2.76; 30.72]	9.49 [0.04; 30.73]	7.02 [4.16; 10.52]
	Adolescents	57.4 [17.9; 92.2]	35.9 [15.0; 59.2]	46.4 [27.6; 65.7]	1.89 [1.55; 2.27]	1.12 [0.01; 3.59]	2.05 [0.33; 4.98]
	Women with HIV	64.7 [48.3; 79.7]	61.0 [42.7; 78.0]	70.5 [60.7; 79.6]	10.11 [0.13; 31.39]	3.09 [0.68; 6.96]	6.42 [1.89; 13.23]
	Infertile	74.7 [59.9; 87.2]	..	100 [5.5; 89.2]	11.76 [4.33; 22.07]	0.00 [0.07; 3.40]	0.73 [0.04; 4.60]

* These analyses excluded populations with an increased risk of STI (FSW, women with HIV, and women attending an STI clinic)

† These analyses were conducted exclusively on these populations and did not consist of a sub-group analysis. "Pregnant women" and "Women with HIV" are not mutually exclusive

The proportion of asymptomatic CT, NG, and TV infections (excluding populations with an increased risk of STIs) was significantly different across regions (p = 0.048, p = 0.019, and p = 0.039, respectively for each pathogen). Africa presented the highest proportion of asymptomatic infections (CT: 68.6% [56.4; 79.8], 14 data points, 12 368 participants; NG: 67.2% [51.0; 81.9], 13 data points, 7 681 participants; TV: 64.9% [52.2; 76.7], 12 data points, 6 152 participants). Latin America had the lowest proportion of asymptomatic women infected with CT (30.9% [11.2; 54.8], five data points, 2 850 participants) and with NG (14.0% [0.0; 67.5], two data points, 1 546 participants). The lowest proportion of asymptomatic TV infection was found in Asia (17.3% [0.0; 49.5], four data points, 2 606 participants).

When stratifying by country income level, the proportions of asymptomatic cases were the highest in middle-income countries (CT: 63.2% [50.2; 75.1], 11 data points, 14 887 participants; NG: 60.9% [41.1; 79.2], 14 data points, 10 793 participants; TV: 61.7% [45.4; 77.1], 17 data points, 6 688 participants), and the lowest in low-income countries (CT: 55.5% [37.3; 73.1], 11 data points, 11 381 participants; NG: 37.7% [18.4; 58.8], seven data points, 6 157 participants; TV: 45.4% [29.7; 61.6], six data points, 4 136 participants). The proportion of asymptomatic infected women was also the highest in rural settings (CT: 71.6% [55.6; 85.3], ten data points, 9 150 participants; NG: 70.4% [46.3; 90.3], 9 data points, 4 464 participants; TV: 75.0% [49.8; 94.0], six data points, 2 619 participants), and the lowest in urban settings (CT: 55.6% [43.8; 67.1], 20 data points, 15 276 participants; NG: 44.4% [26.5; 62.9], 11 data points, 12 124 participants; TV: 58.9% [46.5; 70.8], 15 data points, 7 544 participants).

Analyses on specific populations showed that, in women with HIV, the proportion of asymptomatic CT, NG, and TV infections exceeded 50% (CT: 64.7% [48.3; 79.7], three data points, 739 participants; NG: 61.0% [42.7; 78.0], three data points, 739 participants; TV: 70.5% [60.7; 79.6], four data points, 965 participants). In FSWs, those proportions were below 50% (CT: 45.5% [24.8; 67.0], five data points, 1 890 participants; NG: 38.1% [3.1; 82.9], three data points, 1 051 participants; TV: 26.7% [19.6; 34.5], two data points, 546 participants).

The prevalence of asymptomatic women infected with CT, NG, or TV was 4.70 per 100 women [3.39; 6.20] (33 data points, 26 268 participants), 3.11 [1.34; 5.54] (21 data points, 16 950 participants) and 5.98 [3.46; 9.12] (23 data points, 10 824 participants), respectively, excluding populations with an increased risk of STI infection.

The prevalence of asymptomatic NG and TV infections differed significantly across regions (p<0.001). The highest prevalence was found in Africa and Oceania (NG: 5.67 [2.15; 10.66] and 3.12 [0.00; 12.25], TV: 9.06 [5.71; 13.07] and 12.77 [0.06; 40.12], respectively) and the lowest in Asia and Latin America (NG: 0.13 [0.03; 0.28] and 0.12 [0.00; 1.16], TV: 0.08 [0.00; 0.31] and 4.54 [0.52; 11.9], respectively). A similar pattern was observed for CT, but there was weak evidence of this difference (p = 0.068).

For NG only, the prevalence of asymptomatic infection was significantly higher in middle-income countries versus low-income (5.21 [1.77; 10.22] vs 0.57 [0.17; 1.17], p = 0.002) and was nearly significantly different between rural and urban settings (6.80 [1.39; 15.60] vs 1.33 [0.29; 3.02], p = 0.059).

Relative to the other key populations, the prevalence of asymptomatic CT, NG, and TV was low among adolescents (CT: 1.89 [1.55; 2.27]; NG: 1.12 [0.01; 3.59]; TV: 2.05 [0.33; 4.98]) and high among FSWs (CT: 13.6 [2.76; 30.72]; NG: 9.49 [0.04; 30.73]; TV:7.02 [4.16; 10.52]).

**Fig 4:**
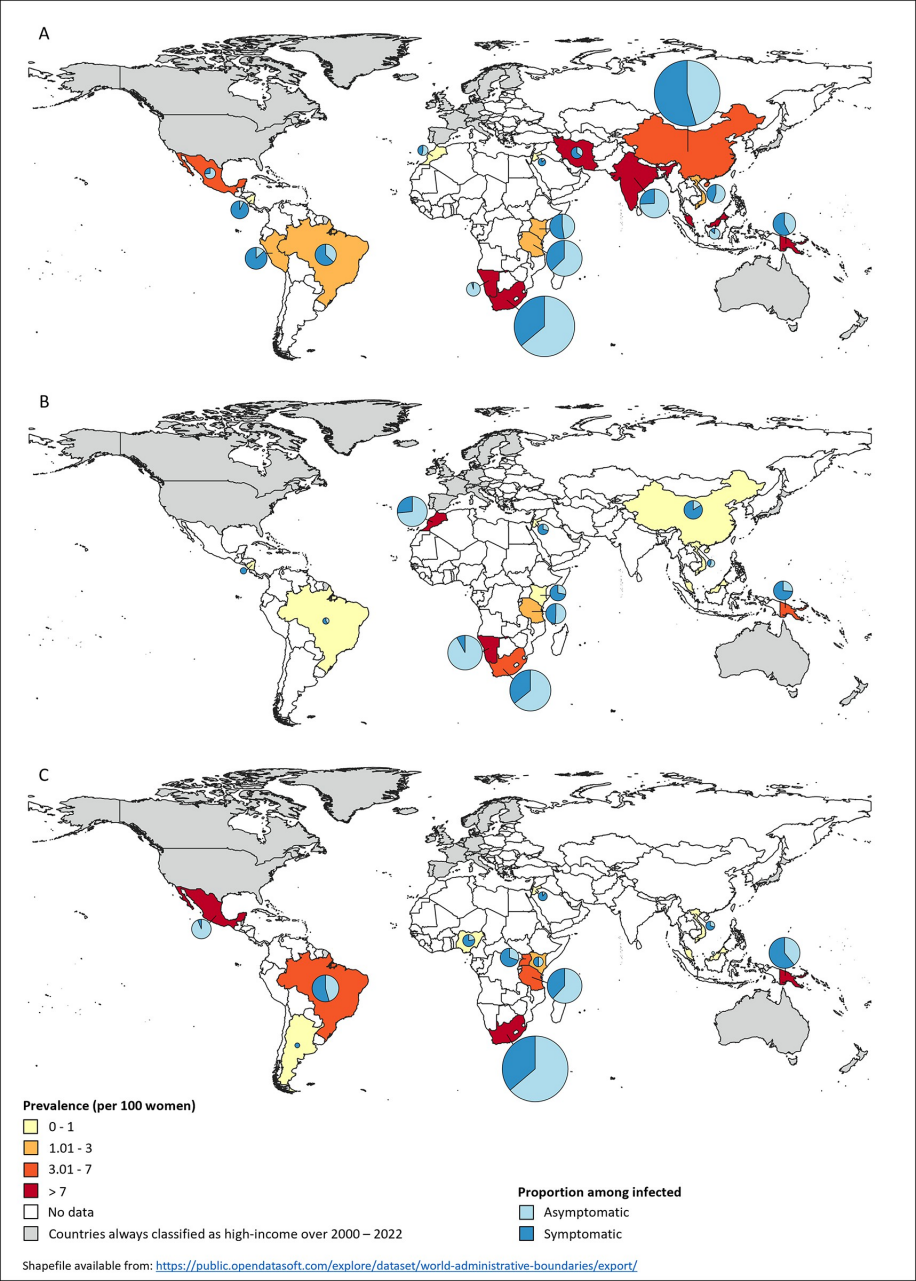
Maps of asymptomatic CT (A), NG (B) and TV (C) infections in women, in LMICs, by country.

Proportion and prevalence by country are presented in Fig 4 and S6 Table.

After excluding studies with a high risk of bias, our sensitivity analyses showed no significant change in our findings (S7–S9 Tables). Results of the meta-regression showed that the continent and the country income level explained a substantial part of the variation in our estimates (S10 Table). Results from the Funnel plots and Egger’s tests showed no evidence of publication bias (S1–S3 Fig).

## Discussion

Our study reveals a significant public health challenge: more than half of the women infected with CT, NG, and TV were asymptomatic in LMICs. The prevalence of asymptomatic infections ranged from 3 to 6%. When cumulating the prevalence of each of pathogen, accounting for the rarity of co-infections, [15, 16] the prevalence could reach up to 13%. These high proportions and prevalence of asymptomatic infections are particularly worrisome since those women remain untreated and thus may develop serious complications. Additionally, they constitute an unseen reservoir that continues to fuel the STI epidemic.

Several hypotheses can be proposed to explain these high estimates. First, women may have difficulties in distinguishing physiological from abnormal vaginal discharge. For instance, in Tanzania, a study found that more than two third of women with abnormal discharge did not report any symptoms [17]. In rural areas of Madagascar, anthropologists reported that genital symptoms are thought to be normal [18] and women may not report them, even after probing.

Alternatively, social norms may prevent women from declaring their symptoms. They may correctly identify genital symptoms but may be unwilling to communicate them to healthcare professionals due to the stigma attached to such symptoms or fear of being judged [19].

The notably high proportions of asymptomatic CT, NG, and TV infections in Africa are concerning. STIs are associated with an increased risk of transmitting and acquiring other STIs or HIV and Africa bears amongst the highest STI and HIV prevalence in the world [2, 20].The wide use of syndromic approach in Africa means that asymptomatic women are often left undiagnosed and untreated, posing a risk of transmission to their partners and contributing to adverse health outcomes.

We observed a high proportion of asymptomatic STIs among women with HIV. While the limited number of studies limits definitive conclusions, similar findings have been reported among men with HIV who have sex with men [21]. In this study, detectable plasma HIV RNA, social factors, and sexual practices were associated with asymptomatic STIs. Further research could also explore the potential immunological mechanisms underlying this phenomenon.

Our study revealed an elevated prevalence of asymptomatic STIs among FSWs. This reflects their increased susceptibility to STIs explained by their frequent engagement in sexual activities with multiple partners. However, we observed a relatively lower proportion of asymptomatic women, which could be explained by FSWs’ increased awareness and recognition of STI symptoms [22].

We found a low prevalence of asymptomatic STIs among adolescents, possibly due to the lower STI prevalence in this group and the inclusion of sexually inactive participants in some studies. However, despite the lower prevalence, adolescents remain vulnerable to STI acquisition due to their sexual behavior and physiological susceptibility [23]. Implementing comprehensive sexual education programs during early adolescence could effectively maintain a low STI occurrence in this key population [24].

This study has several limitations. Firstly, the majority of the studies were conducted in healthcare facilities which may introduce bias, as women could attend these facilities because of genitourinary symptoms, potentially underrepresenting asymptomatic cases compared to the general population. Consequently, while our estimates are already substantial, the true proportion and prevalence could be even higher. Furthermore, our search yielded only six articles conducted in community settings, indicating a gap in community-based research. Such studies are pivotal for accurately assessing the proportion and prevalence of asymptomatic STIs in the general population. Another limitation pertains to the varying number of symptoms used to determine symptomatic status, ranging from one to fourteen. Studies examining more symptoms were more likely to identify symptomatic cases and fewer asymptomatic cases. However, our comparison of estimates from studies assessing one to four symptoms versus those assessing five or more symptoms did not show any significant differences. Establishing a uniform definition in future research could potentially reduce the heterogeneity among different studies.

Another limitation of our review is that 39 studies appeared to have collected the data we needed but we were unable to obtain these figures. However, we did not find any temporal or spatial pattern among them. In addition, due to our language restrictions, ten studies conducted in Latin America were excluded although they seemed to be relevant.

Nevertheless, our study has several strengths. To the best of our knowledge, this is the first meta-analysis providing comprehensive estimated on asymptomatic STIs in LMICs among women. Our strict inclusion and exclusion criteria, particularly regarding pathogen identification techniques minimized the risk of including false positive cases and ensured that the study’s findings were based on high-quality and reliable data. We also provided estimates for specific populations of pregnant women, FSWs, adolescents, women with HIV, and infertile women. Further research is needed. Within this large reservoir of asymptomatic STIs, it is important to estimate what percentage of women will develop complications such as PID as well as what percentage will spontaneously clear the pathogen [25] It is also critical to identify characteristics associated with developing complications or sequelae in order to treat those women at an early stage.

We showed that the prevalence and proportion of asymptomatic STIs among women in LMICs represent a significant public health concern. An alternative to the syndromic management could be the development of an easy-to-use and affordable point-of-care test (POCT) that would rapidly and accurately detect STIs [26]. A POCT to screen women with suspected STIs among asymptomatic women would be more cost-effective than POCT testing for each pathogen on all of them. Currently, a POCT detecting inflammation caused by any STI is being tested [27]. In addition to providing confidential healthcare services and destigmatizing STIs as defined in the global health strategies on STIs by the WHO for 2022–2030, [28] such a device could be used by health care providers to screen asymptomatic women in order to prevent adverse consequences for reproductive and overall health.

### Data sharing

The study protocol is available on PROSPERO (CRD42022286673). The equations for searching the databases are given in S1 Text. Countries’ classifications into income levels are publicly available from the World Bank [11]. Data extracted from the studies (deidentified data) and the data dictionary are publicly available with no restriction at https://doi.org/10.57745/C61VFC.

## Supporting information

S1 Text Search terms for citation selection. (DOCX)

S2 Text Formulae of the calculation of proportion and prevalence of asymptomatic infections. **Formula:** Calculation of prevalence and proportion of asymptomatic infections.
(DOCX)

S3 Text References of included articles. (DOCX)

S1 Table PRISMA checklist. (DOCX)

S2 Table List and characteristics of included articles. (DOCX)

S3 Table Proportion and prevalence of asymptomatic CT infections: Number of studies, number of participants, and I^2^. (DOCX)

S4 Table Proportion and prevalence of asymptomatic NG infections: Number of studies, number of participants, and I^2^. (DOCX)

S5 Table Proportion and prevalence of asymptomatic TV infections: Number of studies, number of participants, and I^2^. (DOCX)

S6 Table Sensitivity analysis–proportion and prevalence estimates of asymptomatic CT infections excluding high risk of bias studies. (DOCX)

S7 Table Sensitivity analysis–proportion and prevalence estimates of asymptomatic NG infections excluding high risk of bias studies. (DOCX)

S8 Table Sensitivity analysis–proportion and prevalence estimates of asymptomatic TV infections excluding high risk of bias studies. (DOCX)

S9 Table Proportion and prevalence of asymptomatic CT, NG, and TV by country (data used for the map). (DOCX)

S10 Table Meta-regression estimates and 95% CI for the proportion and the prevalence of asymptomatic CT (A), NG (B), and TV (C).
(DOCX)

S1 Fig Funnel plot and Egger’s test for the proportion of asymptomatic CT. (DOCX)

S2 Fig Funnel plot and Egger’s test for the proportion of asymptomatic NG. (DOCX)

S3 Fig Funnel plot and Egger’s test for the proportion of asymptomatic TV. (DOCX)
