# Supplementary material for: Asymptomatic infections with Chlamydia trachomatis, Neisseria gonorrhoeae, and Trichomonas vaginalis among women in low- and middle-income countries: A systematic review and meta-analysis
Source: PLOS Glob Public Health. 2024 May 23;4(5):e0003226. doi: 10.1371/journal.pgph.0003226 (PMC11115196; doi:10.1371/journal.pgph.0003226)
Supplement: S1 Text — (DOCX) [file pgph.0003226.s001.docx]

**S1 Text: Search terms for citation selection**

**PubMed**

((women[MH] OR female[MH])
OR
(“woman”[TW] OR “women*”[TW] OR “female*”[TW] OR (“adolescent*”[TW] AND “female*”[TW]) OR “girl*”[TW]))

AND

((“asymptomatic infections”[MH] OR “asymptomatic diseases”[MH])
OR
(asymptomatic*[TW] OR “not symptomatic” [TW] OR “absence of symptom*” [TW] OR “without symptom*” [TW] OR “no symptom*” [TW] OR “no sign*”[TW] OR “absence of sign*”[TW] OR “silent infection*”[TW] OR “presymptomatic*” [TW] OR “pre-symptomatic*”[TW]))

AND

((“chlamydia trachomatis”[MH] OR “chlamydia infections”[MH])
OR
(“chlamydia”[TW] OR “chlamydiae”[TW] OR “chlamydia trachomatis” [TW] OR “C. trachomatis” [TW] or (“Chlamydia”[TW] AND “trachomatis” [TW]))

OR

("Neisseria gonorrhoeae"[MH] OR "Gonorrhea"[MH])
OR
("gonorrhea"[TW] OR "gonorrhoea"[TW] OR "gonorrhoeae"[TW] OR “Neisseria gonorrhoeae” [TW] OR “N. gonorrhoeae” [TW] OR (“Neisseria” [TW] AND “gonorrhoeae”[TW]) OR "gonococcal"[TW])

OR

(“Trichomonas vaginalis”[MH] OR “Trichomonas vaginitis”[MH] OR “Trichomonas infections”[MH])
OR
(trichomoniases[TW] OR trichomoniasis[TW] OR “Trichomona*”[TW] OR “Trichomonas vaginalis” [TW] OR “T. vaginalis”[TW] OR (“Trichomonas”[TW] AND “vaginalis”[TW]) OR “Trichomonas vaginali” [TW] OR “T. vaginali”[TW] OR (“Trichomonas”[TW] AND “vaginali”[TW]) OR “trichomonas vaginitides”[TW] OR (“Trichomonas” AND “vaginitides”[TW]))
)

AND

( "Afghanistan"[TW] OR "Albania"[TW] OR "Algeria"[TW] OR “American Samoa”[TW] OR "Angola"[TW] OR "Antigua"[TW] OR "Argentina"[TW] OR "Armenia"[TW] OR "Azerbaijan"[TW] OR "Bangladesh"[TW] OR "Barbuda"[TW] OR "Belarus"[TW] OR "Belize"[TW] OR "Benin"[TW] OR "Bhutan"[TW] OR "Bolivia"[TW] OR "Bosnia"[TW] OR "Herzegovina"[TW] OR "Hercegovina"[TW] OR "Bosnia-Herzegovina"[TW] OR "Botswana"[TW] OR "Brazil"[TW] OR "Bulgaria"[TW] OR "Burkina Faso"[TW] OR "Burundi"[TW] OR "Cabo Verde"[TW] OR "Cape Verde"[TW] OR "Cambodia"[TW] OR "Cameroon"[TW] OR "Central African Republic"[TW] OR "Chad"[TW] OR "Chili"[TW] OR "China"[TW] OR "Colombia"[TW] OR "Comoros"[TW] OR "Costa Rica"[TW] OR "Cote d’Ivoire"[TW] OR "Ivory Coast"[TW] OR "Croatia"[TW] OR "Cuba"[TW] OR "Czech Republic"[TW] OR "Czechia"[TW] OR "Democratic republic of the Congo"[TW] OR "Congo"[TW] OR "Zaire"[TW] OR "DRC"[TW] OR "Democratic People’s Republic Korea"[TW] OR "North Korea"[TW] OR "Djibouti"[TW] OR "Dominica"[TW] OR "Dominican Republic"[TW] OR "Ecuador"[TW] OR "Egypt"[TW] OR "El Salvador"[TW] OR "Salvador"[TW] OR "Eritrea"[TW] OR "Eswatini"[TW] OR "Swaziland"[TW] OR "Equatorial Guinea"[TW] OR "Estonia"[TW] OR "Ethiopia"[TW] OR "Fiji"[TW] OR "Gabon"[TW] OR "Gambia"[TW] OR "Georgia"[TW] OR "Ghana"[TW] OR "Gibraltar"[TW] OR "Grenada"[TW] OR "Guatemala"[TW] OR "Guinea"[TW] OR "Guinea-Bissau"[TW] OR "Guyana"[TW] OR "Haiti"[TW] OR "Honduras"[TW] OR "Hungary"[TW] OR "India"[TW] OR "Indonesia"[TW] OR "Iran"[TW] OR "Iraq"[TW] OR "Jamaica"[TW] OR "Jordan"[TW] OR "Kazakhstan"[TW] OR "Kenya"[TW] OR "Kiribati"[TW] OR "Kosovo"[TW] OR "Kyrgyzstan"[TW] OR "Kirghizia"[TW] OR "Kyrgyz Republic"[TW] OR "Kirgizstan"[TW] OR "Kirghizstan"[TW] OR "Kyrghizstan"[TW] OR "Lao PDR"[TW] OR "Laos"[TW] OR "Lao People*"[TW] OR "Latvia"[TW] OR "Lebanon"[TW] OR "Lesotho"[TW] OR "Liberia"[TW] OR "Libya"[TW] OR "Lithuania"[TW] OR "Madagascar"[TW] OR "Malawi"[TW] OR "Malaysia"[TW] OR "Maldives"[TW] OR "Mali"[TW] OR "Malta"[TW] OR "Marshall Islands"[TW] OR "Mauritania"[TW] OR "Mauritius"[TW] OR "Mexico"[TW] OR "Micronesia"[TW] OR "Moldova"[TW] OR "Mongolia"[TW] OR "Montenegro"[TW] OR "Morocco"[TW] OR "Mozambique"[TW] OR "Myanmar"[TW] OR "Namibia"[TW] OR "Nauru"[TW] OR "Nepal"[TW] OR "Nicaragua"[TW] OR "Niger"[TW] OR "Nigeria"[TW] OR "Northern Mariana Islands"[TW] OR “North Macedonia"[TW] OR "Macedonia"[TW] OR "Oman"[TW] OR "Pakistan"[TW] OR "Panama"[TW] OR "Papua New Guinea"[TW] OR "PNG"[Title/Abstract] OR "Paraguay"[TW] OR "Paulu"[TW] OR "Peru"[TW] OR "Philippines"[TW] OR "Poland"[TW] OR "Porto Rico"[TW] OR "Republic of the Congo"[TW] OR "Romania"[TW] OR "Russian Federation"[TW] OR "Russia"[TW] OR "Rwanda"[TW] OR "Samoa"[TW] OR "Sao Tome"[TW] OR "Principe"[TW] OR "Saudi Arabia"[TW] OR "Senegal"[TW] OR "Serbia"[TW] OR "Sierra Leone"[TW] OR "Solomon Islands"[TW] OR "Somalia"[TW] OR "South Africa"[TW] OR "South Sudan"[TW] OR "Sri Lanka"[TW] OR "St Kitts"[TW] OR "Saint Kitts"[TW] OR "Nevis"[TW] OR "St Lucia"[TW] OR "Saint Lucia"[TW] OR "St Vincent"[TW] OR "the Grenadines"[TW] OR "Saint Vincent"[TW] OR "Slovakia"[TW] OR "Sudan"[TW] OR "Suriname"[TW] OR "Syria"[TW] OR "Syrian Arab Republic"[TW] OR "Tajikistan"[TW] OR "Tadjikistan"[TW] OR "Tadzhikistan"[TW] OR "Tanzania"[TW] OR "Thailand"[TW] OR "Timor-Leste"[TW] OR "Timor Leste"[TW] OR "Tobago"[TW] OR "Togo"[TW] OR "Tonga"[TW] OR "Trinidad"[TW] OR "Tunisia"[TW] OR "Turkey"[TW] OR "Turkmenistan"[TW] OR "Tuvalu"[TW] OR "Uganda"[TW] OR "Ukraine"[TW] OR "Uruguay"[TW] OR "Uzbekistan"[TW] OR "Vanuatu"[TW] OR "Venezuela"[TW] OR "Vietnam"[TW] OR "West Bank"[TW] OR "Gaza"[TW] OR "Palestine"[TW] OR "Yemen"[TW] OR "Zambia"[TW] OR "Zimbabwe"[TW]

)

AND (2000:2022[pdat])

**Scopus**

TITLE-ABS-KEY ( "woman" OR "women*" OR "female*" OR ( "adolescent*" AND "female*" ) OR "girl*" )

AND

TITLE-ABS-KEY ( "asymptomatic*" OR "not symptomatic" OR "absence of symptom*" OR “without symptom*” OR "no symptom*" OR "no sign*" OR "absence of sign*" OR "presymptomatic*" OR "pre-symptomatic*" )

AND

TITLE-ABS-KEY ( "Chlamydia trachomatis" OR "Chlamydia infection*" OR "chlamydia" OR "chlamydiae" OR "C. trachomatis" OR ( "Chlamydia" AND "trachomatis" )

OR

"Neisseria gonorrhoeae" OR "gonorrhea " OR "gonorrhoea" OR "gonorrhoeae" OR "N. gonorrhoeae" OR ( "Neisseria" AND "gonorrhoeae" ) OR "gonococcal"

OR

"Trichomonas vaginalis" OR "Trichomonas vaginitis" OR "T. vagilanis" OR ( "Trichomonas" AND "vaginalis" ) OR "Trichomonas vaginali" OR "T. vaginali" OR ( "Trichomonas" AND "Vaginali" ) OR ( "Trichomonas vaginitides" ) OR ( "Trichomonas" AND "vaginitides" ) )

AND

TITLE-ABS-KEY ( "Afghanistan" OR "Albania" OR "Algeria" OR "American Samoa" OR "Angola" OR "Antigua" OR "Argentina" OR "Armenia" OR "Azerbaijan" OR "Bangladesh" OR "Barbuda" OR "Belarus" OR "Belize" OR "Benin" OR "Bhutan" OR "Bolivia" OR "Bosnia" OR "Herzegovina" OR "Hercegovina" OR "Bosnia-Herzegovina" OR "Botswana" OR "Brazil" OR "Bulgaria" OR "Burkina Faso" OR "Burundi" OR "Cabo Verde" OR "Cape Verde" OR "Cambodia" OR "Cameroon" OR "Central African Republic" OR "Chad" OR "Chile" OR "China" OR "Colombia" OR "Comoros" OR "Costa Rica" OR "Cote d’Ivoire" OR "Ivory Coast" OR "Croatia" OR "Cuba" OR "Czech Republic" OR "Czechia" OR "Democratic republic of the Congo" OR "Congo" OR "Zaire" OR "DRC" OR "Democratic People’s Republic Korea" OR "North Korea" OR "Djibouti" OR "Dominica" OR "Dominican Republic" OR "Ecuador" OR "Egypt" OR "El Salvador" OR "Equatorial Guinea" OR "Salvador" OR "Eritrea" OR "Estonia" OR "Eswatini" OR "Swaziland" OR "Ethiopia" OR "Fiji" OR "Gabon" OR "Gambia" OR "Georgia" OR "Ghana" OR "Gibraltar" OR "Grenada" OR "Guatemala" OR "Guinea" OR "Guinea-Bissau" OR "Guyana" OR "Haiti" OR "Honduras" OR "Hungary" OR "India" OR "Indonesia" OR "Iran" OR "Iraq" OR "Jamaica" OR "Jordan" OR "Kazakhstan" OR "Kenya" OR "Kiribati" OR "Kosovo" OR "Kyrgyzstan" OR "Kirghizia" OR "Kyrgyz Republic" OR "Kirgizstan" OR "Kirghizstan" OR "Kyrghizstan" OR "Lao PDR" OR "Laos" OR "Lao People*" OR "Latvia" OR "Lebanon" OR "Lesotho" OR "Liberia" OR "Libya" OR "Lithuania" OR "Madagascar" OR "Malawi" OR "Malaysia" OR "Maldives" OR "Mali" OR "Malta" OR "Marshall Islands" OR "Mauritania" OR "Mauritius" OR "Mexico" OR "Micronesia" OR "Moldova" OR "Mongolia" OR "Montenegro" OR "Morocco" OR "Mozambique" OR "Myanmar" OR "Namibia" OR "Nauru" OR "Nepal" OR "Nicaragua" OR "Niger" OR "Nigeria" OR "Northern Mariana Islands" OR "North Macedonia" OR "Macedonia" OR "Oman" OR "Pakistan" OR "Panama" OR "Papua New Guinea" OR "Paraguay" OR "Paulu" OR "Peru" OR "Philippines" OR "Poland" OR "Porto Rico" OR "Republic of the Congo" OR "Romania" OR "Russian Federation" OR "Russia" OR "Rwanda" OR "Samoa" OR "Sao Tome" OR "Principe" OR "Saudi Arabia" OR "Senegal" OR "Serbia" OR "Sierra Leone" OR "Slovakia" OR "Solomon Islands" OR "Somalia" OR "South Africa" OR "South Sudan" OR "Sri Lanka" OR "Saint Kitts" OR "St Kitts" OR "Nevis" OR "St Lucia" OR "Saint Lucia" OR "St Vincent" OR "the Grenadines" OR "Saint Vincent" OR "Sudan" OR "Suriname" OR "Syria" OR "Syrian Arab Republic" OR "Tajikistan" OR "Tadjikistan" OR "Tadzhikistan" OR "Tanzania" OR "Thailand" OR "Timor-Leste" OR "Timor Leste" OR "Tobago" OR "Togo" OR "Tonga" OR "Trinidad" OR "Tunisia" OR "Turkey" OR "Turkmenistan" OR "Tuvalu" OR "Uganda" OR "Ukraine" OR "Uruguay" OR "Uzbekistan" OR "Vanuatu" OR "Venezuela" OR "Vietnam" OR "West Bank" OR "Gaza" OR "Palestine" OR "Yemen" OR "Zambia" OR "Zimbabwe" )

AND

PUBYEAR > 1999 AND PUBYEAR < 2023

**Web of Science**

TS=("woman" OR "women*" OR "female*" OR ("adolescent*" AND "female*") OR "girl*")

AND

TS=( "asymptomatic*" OR "not symptomatic" OR "absence of symptom*" OR "without symptom*" OR "no symptom*" OR "no sign*" OR "absence of sign*" OR "silent infection*" OR "presymtomatic*" OR "pre-symptomatic*")

AND

(TS=(“chlamydia trachomatis" or “chlamydia infection*" or "chlamydia" or "chlamydiae" or "c. trachomatis" or ("chlamydia" AND "trachomatis"))

OR

TS=("Neisseria gonorrhoeae" OR "gonorrhea" OR "gonorrhoea" OR "gonorrhoeae" OR "N. gonorrhoeae" OR ("Neisseria" AND "gonorrhoeae") OR "gonococcal")

OR

TS=("Trichomonas vaginalis" OR "Trichomonas vaginitis" OR "T. vagilanis" OR ("Trichomonas" AND "vaginalis") OR "Trichomonas vaginali" OR "T. vaginali" OR ("Trichomonas" AND "Vaginali") OR "Trichomonas vaginitides" OR ( "Trichomonas" AND "vaginitides" )))

AND

ALL=( ( "Afghanistan" OR "Albania" OR "Algeria" OR "American Samoa" OR "Angola" OR "Antigua" OR "Argentina" OR "Armenia" OR "Azerbaijan" OR "Bangladesh" OR "Barbuda" OR "Belarus" OR "Belize" OR "Benin" OR "Bhutan" OR "Bolivia" OR "Bosnia" OR "Herzegovina" OR "Hercegovina" OR "Bosnia-Herzegovina" OR "Botswana" OR "Brazil" OR "Bulgaria" OR "Burkina Faso" OR "Burundi" OR "Cabo Verde" OR "Cape Verde" OR "Cambodia" OR "Cameroon" OR "Central African Republic" OR "Chad" OR "Chile" OR "China" OR "Colombia" OR "Comoros" OR "Costa Rica" OR "Cote d’Ivoire" OR "Ivory Coast" OR "Croatia" OR "Cuba" OR "Czech Republic" OR "Czechia" OR "Democratic republic of the Congo" OR "Congo" OR "Zaire" OR "DRC" OR "Democratic People’s Republic Korea" OR "North Korea" OR "Djibouti" OR "Dominica" OR "Dominican Republic" OR "Ecuador" OR "Egypt" OR "El Salvador" OR "Equatorial Guinea" OR "Salvador" OR "Eritrea" OR "Estonia" OR "Eswatini" OR "Swaziland" OR "Ethiopia" OR "Fiji" OR "Gabon" OR "Gambia" OR "Georgia" OR "Ghana" OR "Gibraltar" OR "Grenada" OR "Guatemala" OR "Guinea" OR "Guinea-Bissau" OR "Guyana" OR "Haiti" OR "Honduras" OR "Hungary" OR "India" OR "Indonesia" OR "Iran" OR "Iraq" OR "Jamaica" OR "Jordan" OR "Kazakhstan" OR "Kenya" OR "Kiribati" OR "Kosovo" OR "Kyrgyzstan" OR "Kirghizia" OR "Kyrgyz Republic" OR "Kirgizstan" OR "Kirghizstan" OR "Kyrghizstan" OR "Lao PDR" OR "Laos" OR "Lao People*" OR "Latvia" OR "Lebanon" OR "Lesotho" OR "Liberia" OR "Libya" OR "Lithuania" OR "Madagascar" OR "Malawi" OR "Malaysia" OR "Maldives" OR "Mali" OR "Malta" OR "Marshall Islands" OR "Mauritania" OR "Mauritius" OR "Mexico" OR "Micronesia" OR "Moldova" OR "Mongolia" OR "Montenegro" OR "Morocco" OR "Mozambique" OR "Myanmar" OR "Namibia" OR "Nauru" OR "Nepal" OR "Nicaragua" OR "Niger" OR "Nigeria" OR "Northern Mariana Islands" OR "North Macedonia" OR "Macedonia" OR "Oman" OR "Pakistan" OR "Panama" OR "Papua New Guinea" OR "Paraguay" OR "Paulu" OR "Peru" OR "Philippines" OR "Poland" OR "Porto Rico" OR "Republic of the Congo" OR "Romania" OR "Russian Federation" OR "Russia" OR "Rwanda" OR "Samoa" OR "Sao Tome" OR "Principe" OR "Saudi Arabia" OR "Senegal" OR "Serbia" OR "Sierra Leone" OR "Slovakia" OR "Solomon Islands" OR "Somalia" OR "South Africa" OR "South Sudan" OR "Sri Lanka" OR "Saint Kitts" OR "St Kitts" OR "Nevis" OR "St Lucia" OR "Saint Lucia" OR "St Vincent" OR "the Grenadines" OR "Saint Vincent" OR "Sudan" OR "Suriname" OR "Syria" OR "Syrian Arab Republic" OR "Tajikistan" OR "Tadjikistan" OR "Tadzhikistan" OR "Tanzania" OR "Thailand" OR "Timor-Leste" OR "Timor Leste" OR "Tobago" OR "Togo" OR "Tonga" OR "Trinidad" OR "Tunisia" OR "Turkey" OR "Turkmenistan" OR "Tuvalu" OR "Uganda" OR "Ukraine" OR "Uruguay" OR "Uzbekistan" OR "Vanuatu" OR "Venezuela" OR "Vietnam" OR "West Bank" OR "Gaza" OR "Palestine" OR "Yemen" OR "Zambia" OR "Zimbabwe" )

The year range was manually selected in Web of Science.
