# Supplementary material for: Asymptomatic infections with Chlamydia trachomatis, Neisseria gonorrhoeae, and Trichomonas vaginalis among women in low- and middle-income countries: A systematic review and meta-analysis
Source: PLOS Glob Public Health. 2024 May 23;4(5):e0003226. doi: 10.1371/journal.pgph.0003226 (PMC11115196; doi:10.1371/journal.pgph.0003226)
Supplement: S2 Text — Formula: Calculation of prevalence and proportion of asymptomatic infections. (DOCX) [file pgph.0003226.s002.docx]

**S2 Text: Formulae of the calculation of proportion and prevalence of asymptomatic infections**

$$Proportion= \frac{Number of women positive for a given STI and asymptomatic}{Number of women positive for that STI}$$

$$Prevalence= \frac{Number of women positive for a given STI and asymptomatic}{Number of women tested for that STI}$$
