# Supplementary material for: Asymptomatic infections with Chlamydia trachomatis, Neisseria gonorrhoeae, and Trichomonas vaginalis among women in low- and middle-income countries: A systematic review and meta-analysis
Source: PLOS Glob Public Health. 2024 May 23;4(5):e0003226. doi: 10.1371/journal.pgph.0003226 (PMC11115196; doi:10.1371/journal.pgph.0003226)
Supplement: S3 Text — (DOCX) [file pgph.0003226.s003.docx]

**S3 Text: References of included articles**

1. Badman SG, Vallely LM, Toliman P, Kariwiga G, Lote B, Pomat W, et al. A novel point-of-care testing strategy for sexually transmitted infections among pregnant women in high-burden settings: results of a feasibility study in Papua New Guinea. BMC Infect Dis. 2016 Jun 6;16(1):250.

2. Bruce E, Bauai L, Masta A, Rooney PJ, Paniu M, Sapuri M, et al. A cross-sectional study of reported symptoms for sexually transmissible infections among female sex workers in Papua New Guinea. Sex Health. 2010 Feb 15;7(1):71–6.

3. Cabeza J, García PJ, Segura E, García P, Escudero F, Rosa SL, et al. Feasibility of Chlamydia trachomatis screening and treatment in pregnant women in Lima, Peru: a prospective study in two large urban hospitals. Sex Transm Infect. 2015 Feb 1;91(1):7–10.

4. Chang SX, Chen KK, Liu XT, Xia N, Xiong PS, Cai YM. Cross-sectional study of asymptomatic Neisseria gonorrhoeae and Chlamydia trachomatis infections in sexually transmitted disease related clinics in Shenzhen, China. Plos One. 2020 Jun 9;15(6):e0234261.

5. Chen H, Luo L, Wen Y, He B, Ling H, Shui J, et al. Chlamydia trachomatis and Human Papillomavirus Infection in Women From Southern Hunan Province in China: A Large Observational Study. Front Microbiol. 2020 May 5;11:827.

6. Chen XS, Yin YP, Liang GJ, Gong XD, Li HS, Poumerol G, et al. Sexually Transmitted Infections Among Female Sex Workers in Yunnan, China. AIDS Patient Care STDs. 2005 Dec;19(12):853–60.

7. Claeys P, Gonzalez C, Gonzalez M, Van Renterghem L, Temmerman M. Prevalence and risk factors of sexually transmitted infections and cervical neoplasia in women’s health clinics in Nicaragua. Sex Transm Infect. 2002 Jun;78(3):204–7.

8. Conde-Ferráez L, Martíez JRC, Ayora-Talavera G, Losa MDRG. Human papillomavirus and Chlamydia trachomatis infection in gyneco-obstetric outpatients from a mexican hospital. Indian J Med Microbiol. 2017;35(1):74–9.

9. Das A, Pathni AK, Narayanan P, George B, Morineau G, Saidel T, et al. High rates of reinfection and incidence of bacterial sexually transmitted infections in a cohort of female sex workers from two Indian cities: need for different STI control strategies? Sex Transm Infect. 2013 Feb 1;89(1):5–10.

10. Davey DLJ, Nyemba DC, Gomba Y, Bekker LG, Taleghani S, DiTullio DJ, et al. Prevalence and correlates of sexually transmitted infections in pregnancy in HIV-infected and- uninfected women in Cape Town, South Africa. PLOS ONE. 2019 Jul 1;14(7):e0218349.

11. Frohlich JA, Abdool Karim Q, Mashego MM, Sturm AW, Abdool Karim SS. Opportunities for treating sexually transmitted infections and reducing HIV risk in rural South Africa. J Adv Nurs. 2007 Nov;60(4):377–83.

12. Ghebremichael M. The Syndromic versus Laboratory Diagnosis of Sexually Transmitted Infections in Resource-Limited Settings. Int Sch Res Not. 2014 Mar 5;2014:e103452.

13. Gokral JS, Mania-Pramanik J, Meherji PK, Mali BN. Introital swab testing for Chlamydia trachomatis in a resource-poor setting: an Indian perspective. Int J Fertil Womens Med. 2005;50(3):140–3.

14. Hazel A, Ponnaluri-Wears S, Davis GS, Low BS, Foxman B. High prevalence of Neisseria gonorrhoeae in a remote, undertreated population of Namibian pastoralists. Epidemiol Infect. 2014 Nov;142(11):2422–32.

15. Hoffman CM, Mbambazela N, Sithole P, Morré SA, Dubbink JH, Railton J, et al. Provision of Sexually Transmitted Infection Services in a Mobile Clinic Reveals High Unmet Need in Remote Areas of South Africa: A Cross-sectional Study. Sex Transm Dis. 2019 Mar;46(3):206–12.

16. Hokororo A, Kihunrwa A, Hoekstra P, Kalluvya SE, Changalucha JM, Fitzgerald DW, et al. High prevalence of sexually transmitted infections in pregnant adolescent girls in Tanzania: a multi-community cross-sectional study. Sex Transm Infect. 2015 Nov;91(7):473–8.

17. Jenab A, Roghanian R, Golbang N, Golbang P, Chamani-Tabriz L. Comparison of three methods of DNA extraction in endocervical specimens for Chlamydia trachomatis infection by spectrophotometry, agarose gel, and PCR. Arch Immunol Ther Exp (Warsz). 2010 Jun;58(3):227–34.

18. Kalsom UA, Suvra B, Zainul R, Siti Norlia O. Prevalence Rates of Chlamydia Trachomatis and Other Sexually Transmitted Organisms in Infertile Couples Attending a Tertiary Medical Centre in Malaysia | IIUM Medical Journal Malaysia [Internet]. [cited 2023 Jun 19]. Available from: https://journals.iium.edu.my/kom/index.php/imjm/article/view/1333

19. Karim S, Bouchikhi C, Banani A, Fatemi HE, Souho T, Erraghay S, et al. Bacterial sexually transmitted infections and syndromic approach: a study conducted on women at Moroccan University Hospital. Germs. 2021 Dec;11(4):544–53.

20. Kerubo E, Laserson KF, Otecko N, Odhiambo C, Mason L, Nyothach E, et al. Prevalence of reproductive tract infections and the predictive value of girls’ symptom-based reporting: findings from a cross-sectional survey in rural western Kenya. Sex Transm Infect. 2016 Jun;92(4):251–6.

21. Lan PT, Lundborg CS, Phuc HD, Sihavong A, Unemo M, Chuc NTK, et al. Reproductive tract infections including sexually transmitted infections: a population-based study of women of reproductive age in a rural district of Vietnam. Sex Transm Infect. 2008 Apr;84(2):126–32.

22. Li C, Tang W, Ho HC, Ong JJ, Zheng X, Sun X, et al. Prevalence of Chlamydia trachomatis Among Pregnant Women, Gynecology Clinic Attendees, and Subfertile Women in Guangdong, China: A Cross-sectional Survey. Open Forum Infect Dis. 2021 Jun 1;8(6):ofab206.

23. Lowe S, Mudzviti T, Mandiriri A, Shamu T, Mudhokwani P, Chimbetete C, et al. Sexually transmitted infections, the silent partner in HIV-infected women in Zimbabwe. South Afr J HIV Med. 2019 Jan 23;20(1):849.

24. Mahafzah AM, Al-Ramahi MQ, Asa’d AM, El-Khateeb MS. Prevalence of sexually transmitted infections among sexually active Jordanian females. Sex Transm Dis. 2008 Jun;35(6):607–10.

25. Mania-Pramanik J, Kerkar S, Sonawane S, Mehta P, Salvi V. Current Chlamydia trachomatis Infection, A Major Cause of Infertility. J Reprod Infertil. 2012 Oct;13(4):204–10.

26. Mbizvo EM, Msuya SE, Stray-Pedersen B, Sundby J, Chirenje ZM, Hussain A. Determinants of reproductive tract infections among asymptomatic women in Harare, Zimbabwe. Cent Afr J Med [Internet]. 2001 [cited 2023 Jun 19];47(3). Available from: https://www.ajol.info/index.php/cajm/article/view/8595

27. Moodley D, Moodley P, Sebitloane M, Soowamber D, McNaughton-Reyes HL, Groves AK, et al. High prevalence and incidence of asymptomatic sexually transmitted infections during pregnancy and postdelivery in KwaZulu Natal, South Africa. Sex Transm Dis. 2015 Jan;42(1):43–7.

28. Mudau M, Peters RP, De Vos L, Olivier DH, J Davey D, Mkwanazi ES, et al. High prevalence of asymptomatic sexually transmitted infections among human immunodeficiency virus-infected pregnant women in a low-income South African community. Int J STD AIDS. 2018;29(4):324–33.

29. Nessa K, Waris SA, Sultan Z, Monira S, Hossain M, Nahar S, et al. Epidemiology and etiology of sexually transmitted infection among hotel-based sex workers in Dhaka, Bangladesh. J Clin Microbiol. 2004 Feb;42(2):618–21.

30. Nessa K, Waris SA, Alam A, Huq M, Nahar S, Chawdhury FAH, et al. Sexually transmitted infections among brothel-based sex workers in bangladesh: high prevalence of asymptomatic infection. Sex Transm Dis. 2005 Jan;32(1):13–9.

31. Obasi AI, Balira R, Todd J, Ross DA, Changalucha J, Mosha F, et al. Prevalence of HIV and Chlamydia trachomatis infection in 15–19-year olds in rural Tanzania. Trop Med Int Health. 2001;6(7):517–25.

32. Peters RPH, Dubbink JH, van der Eem L, Verweij SP, Bos MLA, Ouburg S, et al. Cross-sectional study of genital, rectal, and pharyngeal Chlamydia and gonorrhea in women in rural South Africa. Sex Transm Dis. 2014 Sep;41(9):564–9.

33. Rocha DAP, Filho RAAB, Mariño JM, dos Santos CMB. “Hidden” sexually transmitted infections among women in primary care health services, Amazonas, Brazil. Int J STD AIDS. 2014 Oct;25(12):878–86.

34. Silveira MF, Bruni MP, Stauffert D, Golparian D, Unemo M. Prevalence and risk factors associated with Chlamydia trachomatis, Neisseria gonorrhoeae, and Mycoplasma genitalium among women in Pelotas, Southern Brazil. Int J STD AIDS. 2020 Apr;31(5):432–9.

35. Wangnapi RA, Soso S, Unger HW, Sawera C, Ome M, Umbers AJ, et al. Prevalence and risk factors for Chlamydia trachomatis, Neisseria gonorrhoeae and Trichomonas vaginalis infection in pregnant women in Papua New Guinea. Sex Transm Infect. 2015 May 1;91(3):194–200.

36. Warr AJ, Pintye J, Kinuthia J, Drake AL, Unger JA, McClelland RS, et al. Sexually transmitted infections during pregnancy and subsequent risk of stillbirth and infant mortality in Kenya: a prospective study. Sex Transm Infect. 2019 Feb;95(1):60–6.

37. Adeoye GO, Akande AH. Epidemiology of Trichomonas vaginalis among women in Lagos metropolis, Nigeria. Pak J Biol Sci PJBS. 2007 Jul 1;10(13):2198–201.

38. Bruni MP, Freitas da Silveira M, Stauffert D, Bicca GL de O, Caetano Dos Santos C, da Rosa Farias NA, et al. Aptima Trichomonas vaginalis assay elucidates significant underdiagnosis of trichomoniasis among women in Brazil according to an observational study. Sex Transm Infect. 2019 Mar;95(2):129–32.

39. de Waaij DJ, Dubbink JH, Ouburg S, Peters RPH, Morré SA. Prevalence of Trichomonas vaginalis infection and protozoan load in South African women: a cross-sectional study. BMJ Open. 2017 Oct 8;7(10):e016959.

40. López-Monteon A, Gómez-Figueroa FS, Ramos-Poceros G, Guzmán-Gómez D, Ramos-Ligonio A. Codetection of Trichomonas vaginalis and Candida albicans by PCR in urine samples in a low-risk population attended in a clinic first level in central Veracruz, Mexico. BioMed Res Int. 2013 Aug 29;2013:281892.

41. Mabaso N, Naicker C, Nyirenda M, Abbai N. Prevalence and risk factors for Trichomonas vaginalis infection in pregnant women in South Africa. Int J STD AIDS. 2020 Mar;31(4):351–8.

42. Miranda AE, Pinto VM, Gaydos CA. Trichomonas vaginalis infection among young pregnant women in Brazil. Braz J Infect Dis Off Publ Braz Soc Infect Dis. 2014;18(6):669–71.

43. Mucci MJ, Cuestas ML, Cervetto MM, Landaburu MF, Mujica MT. A prospective observational study of vulvovagintis in pregnant women in Argentina, with special reference to candidiasis. Mycoses. 2016 Jul;59(7):429–35.

44. Paul H, Peter D, Pulimood SA, Abraham OC, Mathai E, Prasad JH, et al. Role of polymerase chain reaction in the diagnosis of Trichomonas vaginalis infection in human immunodeficiency virus-infected individuals from India (South). Indian J Dermatol Venereol Leprol. 2012;78(3):323–7.

45. Råssjö EB, Kambugu F, Tumwesigye MN, Tenywa T, Darj E. Prevalence of sexually transmitted infections among adolescents in Kampala, Uganda, and theoretical models for improving syndromic management. J Adolesc Health. 2006 Mar 1;38(3):213–21.

46. Shehabi AA, Awwad ZM, Al-Ramahi M, Charvalos E, Abu-Qatouseh LF. Detection of *Mycoplasma genitalium* and *Trichomonas vaginalis* Infections in General Jordanian Patients. Am J Infect Dis. 2009 Mar 31;5(1):7–10.

47. Tann CJ, Mpairwe H, Morison L, Nassimu K, Hughes P, Omara M, et al. Lack of effectiveness of syndromic management in targeting vaginal infections in pregnancy in Entebbe, Uganda. Sex Transm Infect. 2006 Aug;82(4):285–9.

48. Tavakoli Oliaee R, Babaei Z, Hatam GR, Tavakoli Kareshk A, Mahmoudvand H, Vafafar A, et al. Considerable Genetic Diversity of Trichomonas vaginalis Clinical Isolates in a Targeted Population in South of Iran. Iran J Parasitol. 2017 Jun;12(2):251–9.
