# Supplementary material for: Asymptomatic infections with Chlamydia trachomatis, Neisseria gonorrhoeae, and Trichomonas vaginalis among women in low- and middle-income countries: A systematic review and meta-analysis
Source: PLOS Glob Public Health. 2024 May 23;4(5):e0003226. doi: 10.1371/journal.pgph.0003226 (PMC11115196; doi:10.1371/journal.pgph.0003226)
Supplement: S1 Table — (DOCX) [file pgph.0003226.s004.docx]

**S1 Table: PRISMA 2020 checklist**

| **Section and Topic** | **Item #** | **Checklist item** | **Page** |
| --- | --- | --- | --- |
| **TITLE** | | |  |
| Title | 1 | Identify the report as a systematic review.  “Asymptomatic infections with Chlamydia trachomatis, Neisseria gonorrhoeae, and Trichomonas vaginalis among women in low- and middle-income countries: A systematic review and meta-analysis” | 1 |
| **ABSTRACT** | | |  |
| Abstract | 2 | See the PRISMA 2020 for Abstracts checklist. | 2, 3 |
| **INTRODUCTION** | | |  |
| Rationale | 3 | Describe the rationale for the review in the context of existing knowledge.  “To date, no study has provided pooled estimates of the burden of asymptomatic infections of the three most common curable STIs.” | 4 |
| Objectives | 4 | Provide an explicit statement of the objective(s) or question(s) the review addresses.  “In this perspective, we conducted a systematic review and a meta-analysis to estimate: (i) the proportion of asymptomatic women among those infected by CT, NG, and TV and, (ii) the prevalence of asymptomatic CT, NG, and TV infections in the female population in LMICs.” | 4 |
| **METHODS** | | |  |
| Eligibility criteria | 5 | Specify the inclusion and exclusion criteria for the review and how studies were grouped for the syntheses.  “We selected studies published between 1 January 2000 and 31 December 2022, in English and French. We included studies which tested for STIs using genital or urine specimens. We kept studies that used NAATs for the diagnosis of CT, NG, and TV. For TV, we also included articles that used InPouch® or performed wet mount microscopy immediately after collection, as these techniques showed fair sensitivity and specificity and remain common detection methods in LMICs.  We selected studies where the investigator actively asked about at least one symptom, or where participants self-reported symptoms. We excluded articles in which authors assumed the absence of symptoms in the entire study population without collecting such information. Finally, we excluded articles when “asymptomatic” was defined as the absence of clinical signs as reported by the healthcare provider rather than by the women as we intended a participant-centred approach.” | 5, 6 |
| Information sources | 6 | Specify all databases, registers, websites, organisations, reference lists and other sources searched or consulted to identify studies. Specify the date when each source was last searched or consulted.  “We searched MEDLINE, Web of Science, and Scopus databases. Search terms combined pathogen and disease terms, terms related to the absence of symptoms and countries ever classified as LMICs by the World Bank between 2000 and 2022 (full search terms in supplementary Text 1). We also screened references for eligible articles. When the estimates were collected but not presented, we contacted the authors up to three times” | 5 |
| Search strategy | 7 | Present the full search strategies for all databases, registers and websites, including any filters and limits used. | S1 Text |
| Selection process | 8 | Specify the methods used to decide whether a study met the inclusion criteria of the review, including how many reviewers screened each record and each report retrieved, whether they worked independently, and if applicable, details of automation tools used in the process.  “BTH, TC, RR, and CF conducted initial screening of titles and abstracts in pairs and inclusion conflicts were resolved through consensus.” | 6 |
| Data collection process | 9 | Specify the methods used to collect data from reports, including how many reviewers collected data from each report, whether they worked independently, any processes for obtaining or confirming data from study investigators, and if applicable, details of automation tools used in the process.  “CF performed data extraction, and BTH, TC, and RR ensured exactness and validity.” | 6 |
| Data items | 10a | List and define all outcomes for which data were sought. Specify whether all results that were compatible with each outcome domain in each study were sought (e.g. for all measures, time points, analyses), and if not, the methods used to decide which results to collect.  “Extracted data included the number of asymptomatic positive women, total women tested, and women positive for a specific STI. ” | 6 |
|  | 10b | List and define all other variables for which data were sought (e.g. participant and intervention characteristics, funding sources). Describe any assumptions made about any missing or unclear information.  “Other variables we extracted were: first author, publication year, study year, country, recruitment site, number and type of reported symptoms, definition of asymptomatic women, rural/urban setting, specific populations (female sex works (FSWs), adolescents, women with HIV, adolescents, infertile women), age range, specimen type, other STI pathogens tested for, and detection techniques.” | 6 |
| Study risk of bias assessment | 11 | Specify the methods used to assess risk of bias in the included studies, including details of the tool(s) used, how many reviewers assessed each study and whether they worked independently, and if applicable, details of automation tools used in the process.  “The risk of bias was assessed using the appraisal tool for cross-sectional studies (AXIS) using >70% of validated items as a cut-off to qualify studies with a low risk of bias.” | 7 |
| Effect measures | 12 | Specify for each outcome the effect measure(s) (e.g. risk ratio, mean difference) used in the synthesis or presentation of results.  “Proportion and prevalence of asymptomatic CT, NG, and TV infections were estimated first for all studies and excluding studies on FSWs, women with HIV, and women attending an STI clinic as they are not representative of the general population and have a higher risk of having an STI. We referred to these groups as “populations with an increased risk of STIs”. Separate estimations were conducted for FSWs, women with HIV as well as other key populations such as pregnant women, adolescents, and infertile women.” | 6 |
| Synthesis methods | 13a | Describe the processes used to decide which studies were eligible for each synthesis (e.g. tabulating the study intervention characteristics and comparing against the planned groups for each synthesis (item #5)).  “We selected studies where the investigator actively asked about at least one symptom, or where participants self-reported symptoms.” | 5 |
|  | 13b | Describe any methods required to prepare the data for presentation or synthesis, such as handling of missing summary statistics, or data conversions.  “Random-effect models were applied for each of the above analyses using the DerSimonian and Laird variance estimator. As some figures were near 0 or 1, the Freeman-Tukey double arcsine transformation was applied.” | 6 |
|  | 13c | Describe any methods used to tabulate or visually display results of individual studies and syntheses.  “Analyses and forest plots were conducted using *meta* and *metafor* packages in R software (version 4.1.1). Proportions and prevalence maps were generated using qGIS (version 3.22).” | 7 |
|  | 13d | Describe any methods used to synthesize results and provide a rationale for the choice(s). If meta-analysis was performed, describe the model(s), method(s) to identify the presence and extent of statistical heterogeneity, and software package(s) used.  “Proportion and prevalence of asymptomatic CT, NG, and TV infections were estimated first for all studies and excluding studies on FSWs, women with HIV, and women attending an STI clinic as they are not representative of the general population and have a higher risk of having an STI. We referred to these groups as “populations with an increased risk of STIs”. Separate estimations were conducted for FSWs, women with HIV as well as other key populations such as pregnant women, adolescents, and infertile women. […] Random-effect models were applied for each of the above analyses using the DerSimonian and Laird variance estimator. As some figures were near 0 or 1, the Freeman-Tukey double arcsine transformation was applied. Statistical heterogeneity was evaluated using the χ² test with Cochran’s Q and I². Analyses and forest plots were conducted using *meta* and *metafor* packages in R software (version 4.1.1).” | 6, 7 |
|  | 13e | Describe any methods used to explore possible causes of heterogeneity among study results (e.g. subgroup analysis, meta-regression).  “Subgroup analyses were performed by region, country income level, rural/urban setting, study period, and the number of symptoms assessed in the study. The role of these study variables in the heterogeneity of the estimates were explored in meta-regressions.” | 7 |
|  | 13f | Describe any sensitivity analyses conducted to assess robustness of the synthesized results.  “We conducted a sensitivity analysis to assess the impact of excluding studies with a high risk of bias on our overall estimates.” | 7 |
| Reporting bias assessment | 14 | Describe any methods used to assess risk of bias due to missing results in a synthesis (arising from reporting biases).  “Publication bias was evaluated by Funnel plots and the Egger test.” | 7 |
| Certainty assessment | 15 | Describe any methods used to assess certainty (or confidence) in the body of evidence for an outcome. | NA |
| **RESULTS** | | |  |
| Study selection | 16a | Describe the results of the search and selection process, from the number of records identified in the search to the number of studies included in the review, ideally using a flow diagram.  “We identified 1113 articles, among which 547 were duplicates. We eliminated 468 articles based on title and abstract and screened 173 full texts and their citations. We found 4 additional relevant citations among selected articles. In total, 177 articles were eligible. Of those, 42 had available and extractable data and 45 reported having data according to our criteria but did not present the numbers. We were able to contact 40 of those 45 authors and retrieved data from six articles. In total, we used data from 48 studies corresponding to 99 data points for both the prevalence and the proportion: 41 for CT, 27 for NG, and 31 for TV (Fig 1, supplementary Table 2). ” | 7, Fig 1 |
|  | 16b | Cite studies that might appear to meet the inclusion criteria, but which were excluded, and explain why they were excluded. | Fig 1 |
| Study characteristics | 17 | Cite each included study and present its characteristics. | S1 Table |
| Risk of bias in studies | 18 | Present assessments of risk of bias for each included study. | S1 Table |
| Results of individual studies | 19 | For all outcomes, present, for each study: (a) summary statistics for each group (where appropriate) and (b) an effect estimate and its precision (e.g. confidence/credible interval), ideally using structured tables or plots. | S1 Table |
| Results of syntheses | 20a | For each synthesis, briefly summarise the characteristics and risk of bias among contributing studies.  “Twenty-one countries were represented in our study: eight in Africa, seven in Asia, five in Latin America, and one in Oceania. Most studies were cross-sectional (44/48, 92%) and few were cohort (2/48, 4%) or randomized controlled trials (2/48, 4%). Almost three-quarters of the studies took place in urban settings (34/48, 71%), 21% (10/48) in rural settings, and 8% (4/48) in mixed settings. Hospitals and antenatal care were the most frequent recruitment sites (14/48, 29% and 11/48, 23%, respectively), followed by primary or secondary health care centres (9/48, 19%), locations where people with a high risk of STI can be found such as FSW venues, HIV centres or STI clinics (8/48, 17%). Six studies performed random sampling within the community (6/48, 13%). Some studies focused on specific populations: 15/48 (31%) were on pregnant women, 5/48 (10%) on FSWs, 4/48 (8%) on adolescents, 4/48 (8%) on women with HIV, and 4/48 (8%) on infertile women exclusively. Studies had enrolled between 48 and 4812 participants, and their age ranged from 10 to 90 years old although this information was unavailable for 14 studies.” | 7 |
|  | 20b | Present results of all statistical syntheses conducted. If meta-analysis was done, present for each the summary estimate and its precision (e.g. confidence/credible interval) and measures of statistical heterogeneity. If comparing groups, describe the direction of the effect.  “The proportion of asymptomatic CT, NG, and TV infections (excluding populations with an increased risk of STIs) was significantly different across regions (p=0.048, p=0.019, and p=0.039, respectively for each pathogen). ” | 9, 15, Table 1, S2–S4 Tables |
|  | 20c | Present results of all investigations of possible causes of heterogeneity among study results.  “Africa presented the highest proportion of asymptomatic infections (CT: 68.6% [56.4–79.8], 14 data points, 12 368 participants; NG: 67.2% [51.0; 81.9], 13 data points, 7 681 participants; TV: 64.9% [52.2; 76.7], 12 data points, 6 152 participants). Latin America had the lowest proportion of asymptomatic women infected with CT (30.9% [11.2–54.8], five data points, 2 850 participants) and with NG (14.0% [0.0–67.5], two data points, 1 546 participants). The lowest proportion of asymptomatic TV infection was found in Asia (17.3% [0.0–49.5], four data points, 2 606 participants).  When stratifying by country income level, the proportions of asymptomatic cases were the highest in middle-income countries (CT: 63.2% [50.2–75.1], 11 data points, 14 887 participants; NG: 60.9% [41.1–79.2], 14 data points, 10 793 participants; TV: 61.7% [45.4–77.1], 17 data points, 6 688 participants), and the lowest in low-income countries (CT: 55.5% [37.3–73.1], 11 data points, 11 381 participants; NG: 37.7% [18.4–58.8], seven data points, 6 157 participants; TV: 45.4% [29.7–61.6], six data points, 4 136 participants). The proportion of asymptomatic infected women was also the highest in rural settings (CT: 71.6% [55.6–85.3], ten data points, 9 150 participants; NG: 70.4% [46.3–90.3], 9 data points, 4 464 participants; TV: 75.0% [49.8–94.0], six data points, 2 619 participants), and the lowest in urban settings (CT: 55.6% [43.8–67.1], 20 data points, 15 276 participants; NG: 44.4% [26.5–62.9], 11 data points, 12 124 participants; TV: 58.9% [46.5–70.8], 15 data points, 7 544 participants). […]” | 10, 16 |
|  | 20d | Present results of all sensitivity analyses conducted to assess the robustness of the synthesized results. | S5–S7 Tables |
| Reporting biases | 21 | Present assessments of risk of bias due to missing results (arising from reporting biases) for each synthesis assessed. | S1–S3 Fig |
| Certainty of evidence | 22 | Present assessments of certainty (or confidence) in the body of evidence for each outcome assessed. | NA |
| **DISCUSSION** | | |  |
| Discussion | 23a | Provide a general interpretation of the results in the context of other evidence.  “Several hypotheses can be proposed to explain these high estimates. First, women may have difficulties in distinguishing physiological from abnormal vaginal discharge. For instance, in Tanzania, a study found that more than two third of women with abnormal discharge did not report any symptoms.[17] In rural areas of Madagascar, anthropologists reported that genital symptoms are thought to be normal[18] and women may not report them, even after probing.  Alternatively, social norms may prevent women from declaring their symptoms. They may correctly identify genital symptoms but may be unwilling to communicate them to healthcare professionals due to the stigma attached to such symptoms or fear of being judged.[19]  The notably high proportions of asymptomatic CT, NG, and TV infections in Africa are concerning. STIs are associated with an increased risk of transmitting and acquiring other STIs or HIV and Africa bears amongst the highest STI and HIV prevalence in the world.[2,20] The wide use of syndromic approach in Africa means that asymptomatic women are often left undiagnosed and untreated, posing a risk of transmission to their partners and contributing to adverse health outcomes.  We observed a high proportion of asymptomatic STIs among women with HIV. While the limited number of studies limits definitive conclusions, similar findings have been reported among men with HIV who have sex with men.[21] In this study, detectable plasma HIV RNA, social factors, and sexual practices were associated with asymptomatic STIs. Further research could also explore the potential immunological mechanisms underlying this phenomenon.  Our study revealed an elevated prevalence of asymptomatic STIs among FSWs, suggesting an important of circulation of STIs in this population likely due to their important number of sexual partners. However, we observed a relatively lower proportion of asymptomatic women, which could be explained by FSWs' increased awareness and recognition of STI symptoms.[22]  We found a low prevalence of asymptomatic STIs among adolescents, possibly due to the lower STI prevalence in this group and the inclusion of sexually inactive participants in some studies. However, despite the lower prevalence, adolescents remain vulnerable to STI acquisition due to their sexual behavior and physiological susceptibility.[23] Implementing comprehensive sexual education programs during early adolescence could effectively maintain a low STI occurrence in this key population.[24]” | 18, 19 |
|  | 23b | Discuss any limitations of the evidence included in the review.  “Firstly, the majority of the studies were conducted in healthcare facilities which may introduce bias, as women could attend these facilities because of genitourinary symptoms, potentially underrepresenting asymptomatic cases compared to the general population. Consequently, while our estimates are already substantial, the true proportion and prevalence could be even higher. Furthermore, our search yielded only six articles conducted in community settings, indicating a gap in community-based research. Such studies are pivotal for accurately assessing the proportion and prevalence of asymptomatic STIs in the general population. Another limitation pertains to the varying number of symptoms used to determine symptomatic status, ranging from one to fourteen. Studies examining more symptoms were more likely to identify symptomatic cases and fewer asymptomatic cases. However, our comparison of estimates from studies assessing one to four symptoms versus those assessing five or more symptoms did not show any significant differences.” | 19 |
|  | 23c | Discuss any limitations of the review processes used.  “Another limitation of our review is that 39 studies appeared to have collected the data we needed but we were unable to obtain these figures. However, we did not find any temporal or spatial pattern among them. In addition, due to our language restrictions, ten studies conducted in Latin America were excluded although they seemed to be relevant.” | 20 |
|  | 23d | Discuss implications of the results for practice, policy, and future research.  “We showed that the prevalence and proportion of asymptomatic STIs among women in LMICs represent a significant public health concern. An alternative to the syndromic management could be the development of an easy-to-use and affordable point-of-care test (POCT) that would rapidly and accurately detect STIs .[26] A POCT to screen women with suspected STIs among asymptomatic women would be more cost-effective than POCT testing for each pathogen on all of them. Currently, a POCT detecting inflammation caused by any STI is being tested.[27] In addition to providing confidential healthcare services and destigmatizing STIs as defined in the global health strategies on STIs by the WHO for 2022–2030,[28] such a device could be used by health care providers to screen asymptomatic women in order to prevent adverse consequences for reproductive and overall health.” | 20 |
| **OTHER INFORMATION** | | |  |
| Registration and protocol | 24a | Provide registration information for the review, including register name and registration number, or state that the review was not registered.  “The study protocol is available on PROSPERO (CRD42022286673).” | 21 |
|  | 24b | Indicate where the review protocol can be accessed, or state that a protocol was not prepared.  “The study protocol is available on PROSPERO (CRD42022286673).” | 21 |
|  | 24c | Describe and explain any amendments to information provided at registration or in the protocol. | No amendments |
| Support | 25 | Describe sources of financial or non-financial support for the review, and the role of the funders or sponsors in the review.  “This work was supported by the European Union (European and Developing Countries Clinical Trials 2 - EDCTP2 n° RIA2020I-3297, https://www.edctp.org/).” | 21 |
| Competing interests | 26 | Declare any competing interests of review authors.  “We declare no competing interests.” | 21 |
| Availability of data, code and other materials | 27 | Report which of the following are publicly available and where they can be found: template data collection forms; data extracted from included studies; data used for all analyses; analytic code; any other materials used in the review.  “the data dictionary are publicly available with no restriction at https://doi.org/10.57745/FNJZRG” | 21 |
