# Supplementary material for: Asymptomatic infections with Chlamydia trachomatis, Neisseria gonorrhoeae, and Trichomonas vaginalis among women in low- and middle-income countries: A systematic review and meta-analysis
Source: PLOS Glob Public Health. 2024 May 23;4(5):e0003226. doi: 10.1371/journal.pgph.0003226 (PMC11115196; doi:10.1371/journal.pgph.0003226)
Supplement: S2 Table — (DOCX) [file pgph.0003226.s005.docx]

**S2 Table: List and characteristics of included articles**

| **Study** | **Country** | **Income level** | **Study design** | **Setting** | **Method for pathogen detection** | **No. women tested** | **No. women positive** | **No. women positive and asymptomatic** | **Proportion of asymptomatic infection (%)** | **Prevalence of asymptomatic infection (per 100 women)** | **Risk of bias** |
| --- | --- | --- | --- | --- | --- | --- | --- | --- | --- | --- | --- |
| ***Chlamydia trachomatis*** | | | | | | | | | | | |
| Badman, 2016 ^1^ | Papua New Guinea (PNG) | LM | Cross-sectional | Urban | Molecular (PCR) | 125 | 25 | 19 | 76.0 | 15.20 | Low |
| Bruce, 2009 ^2^ | PNG | L | Cross-sectional | Urban | Molecular (PCR) | 129 | 30 | 3 | 10.0 | 2.33 | Low |
| Cabeza, 2015 ^3^ | Peru | UM | Cross-sectional | Urban | Molecular (PCR) | 600 | 60 | 8 | 13.3 | 1.33 | Low |
| Chang, 2020 ^4^ | China | UM | Cross-sectional | Urban | Molecular (PCR) | 4812 | 404 | 138 | 34.2 | 2.87 | High |
| Chen, 2020 ^5^ | China | UM | Cross-sectional | NS | Molecular (PCR) | 5006 | 44 | 44 | 100 | 0.88 | Low |
| Chen, 2005 ^6^ | China | LM | Cross-sectional | Urban | Molecular (PCR) | 505 | 296 | 228 | 77.0 | 45.15 | Low |
| Claeys 2002 ^7^ | Nicaragua | L | Cross-sectional | Urban | Molecular (PCR) | 1185 | 40 | 3 | 7.5 | 0.25 | Low |
| Conde-Ferráez, 2017 ^8^ | Mexico | UM | Cross sectional | Urban | Molecular (PCR) | 233 | 15 | 11 | 73.3 | 4.72 | High |
| Das, 2013 ^9^ | India | LM | Cohort | Urban | Molecular (PCR) | 417 | 47 | 18 | 38.3 | 4.32 | Low |
| Davey, 2019 ^10^ * | South Africa | UM | Cross-sectional | Urban | Molecular (Xpert®) | 135 | 28 | 19 | 67.9 | 14.07 | Low |
| Davey, 2019 ^10^ † | South Africa | UM | Cross-sectional | Urban | Molecular (Xpert®) | 107 | 21 | 10 | 47.6 | 9.35 | Low |
| Frohlich, 2007 ^11^ | South Africa | LM | Cross-sectional | Rural | Molecular (PCR) | 48 | 4 | 4 | 100 | 8.33 | Low |
| Frohlich, 2007 ^11^ | South Africa | LM | Cross-sectional | Rural | Molecular (PCR) | 226 | 20 | 20 | 100 | 8.85 | Low |
| Ghebremichael, 2014 ^12^ | Tanzania | L | Cross-sectional | Urban | Molecular (PCR) | 1440 | 2 | 1 | 50.0 | 0.07 | Low |
| Gokral, 2005 ^13^ | India | L | Cross-sectional | Urban | Molecular (PCR) | 100 | 34 | 29 | 85.3 | 29.00 | High |
| Hazel, 2014 ^14^ | Namibia | UM | Cross-sectional | Rural | Molecular (PCR) | 209 | 24 | 23 | 95.8 | 11.00 | Low |
| Hoffman, 2019 ^15^ | South Africa | UM | Cross-sectional | Rural | Molecular (PCR) | 251 | 52 | 33 | 63.5 | 13.15 | Low |
| Hokororo, 2015 ^16^ | Tanzania | L | Cross-sectional | Rural | Molecular (PCR) | 403 | 46 | 11 | 23.9 | 2.73 | High |
| Jenab, 2010 ^17^ | Iran | LM | Cross-sectional | Urban | Molecular (PCR) | 80 | 17 | 6 | 35.3 | 7.50 | High |
| Kalsom, 2020 ^18^ | Malaysia | UM | Cross-sectional | Urban | Molecular (PCR) | 137 | 15 | 13 | 86.7 | 9.49 | Low |
| Karim, 2021 ^19^ | Morocco | LM | Cross-sectional | Urban | Molecular (PCR) | 809 | 13 | 7 | 53.8 | 0.87 | High |
| Kerubo, 2016 ^20^ | Kenya | L | Cross-sectional | Rural | Molecular (PCR) | 507 | 13 | 9 | 69.2 | 1.78 | Low |
| Lan, 2008 ^21^ | Vietnam | L | Cross-sectional | Rural | Molecular (PCR) | 1012 | 43 | 24 | 55.8 | 2.37 | Low |
| Li, 2021 ^22^ ‡ | China | UM | Cross-sectional | Urban | Molecular (PCR) | 881 | 46 | 39 | 84.8 | 4.43 | Low |
| Li, 2021 ^22^ § | China | UM | Cross-sectional | Urban | Molecular (PCR) | 595 | 35 | 18 | 51.4 | 3.03 | Low |
| Li, 2021 ^22^ ¶ | China | UM | Cross-sectional | Urban | Molecular (PCR) | 254 | 15 | 9 | 60.0 | 3.54 | Low |
| Lowe, 2019 ^23^ | Zimbabwe | L | Cross-sectional | Urban | Molecular (Xpert®) | 385 | 8 | 6 | 75.0 | 1.56 | High |
| Mahafzah, 2008 ^24^ | Jordan | LM | Cross-sectional | Urban | Molecular (PCR) | 1275 | 7 | 1 | 14.3 | 0.08 | High |
| Mania-Pramanik, 2012 ^25^ | India | L | Cross-sectional | Urban | Molecular (PCR) | 174 | 19 | 5 | 26.3 | 2.87 | High |
| Mania-Pramanik, 2012 ^25^ | India | L | Cross-sectional | Urban | Molecular (PCR) | 264 | 30 | 17 | 56.7 | 6.44 | High |
| Mbizvo, 2001 ^26^ | Zimbabwe | L | Cross-sectional | Urban | Molecular (LCR) | 393 | 15 | 8 | 53.3 | 2.04 | Low |
| Moodley, 2015 ^27^ | South Africa | UM | Randomised controlled trials (RCT) | Urban | Molecular (PCR) | 1459 | 259 | 148 | 57.1 | 10.14 | High |
| Mudau, 2018 ^28^ | South Africa | UM | Cross-sectional | Urban | Molecular (Xpert®) | 247 | 91 | 64 | 70.3 | 25.91 | Low |
| Nessa, 2004 ^29^ | Bangladesh | L | Cross-sectional | Urban | Molecular (PCR) | 400 | 174 | 80 | 46.0 | 20.00 | Low |
| Nessa, 2005 ^30^ | Bangladesh | L | Cross-sectional | Urban | Molecular (PCR) | 439 | 77 | 42 | 54.5 | 9.57 | Low |
| Obasi, 2001 ^31^ | Tanzania | L | Cross-sectional | Rural | Molecular (PCR) | 4726 | 113 | 89 | 78.8 | 1.88 | Low |
| Peters, 2014 ^32^ | South Africa | UM | Cross-sectional | Rural | Molecular (PCR) | 589 | 97 | 70 | 72.2 | 11.88 | Low |
| Rocha, 2014 ^33^ | Brazil | UM | Cross-sectional | Urban | Molecular (PCR) | 361 | 23 | 13 | 56.5 | 3.60 | Low |
| Silveira, 2020 ^34^ | Brazil | UM | Cross-sectional | Both | Molecular (PCR) | 498 | 34 | 8 | 23.5 | 1.61 | Low |
| Wangnapi, 2015 ^35^ | PNG | LM | RCT | Both | Molecular (PCR) | 362 | 39 | 8 | 20.5 | 2.21 | Low |
| Warr, 2019 ^36^ | Kenya | L | Cohort | Rural | Molecular (PCR) | 1221 | 65 | 28 | 43.1 | 2.29 | Low |
| ***Neisseria gonorrhoeae*** | | | | | | | | | | | |
| Badman, 2016 ^1^ | PNG | LM | Cross-sectional | Urban | Molecular (PCR) | 125 | 14 | 9 | 64.3 | 11.25 | Low |
| Bruce, 2010 ^2^ | PNG | L | Cross-sectional | Urban | Molecular (PCR) | 129 | 48 | 4 | 8.3 | 1.52 | Low |
| Chang, 2020 ^4^ | China | UM | Cross-sectional | Urban | Molecular (PCR) | 4812 | 43 | 7 | 16.3 | 3.10 | High |
| Chen, 2005 ^6^ | China | LM | Cross-sectional | Urban | Molecular (PCR) | 505 | 191 | 144 | 75.4 | 28.51 | Low |
| Claeys, 2002 ^7^ | Nicaragua | L | Cross-sectional | Urban | Molecular (PCR) | 1185 | 5 | 0 | 0.0 | 0.00 | Low |
| Das, 2013 ^9^ | India | LM | Cohort | Urban | Molecular (PCR) | 417 | 43 | 15 | 34.9 | 2.96 | Low |
| Davey, 2019 ^10^ * | South Africa | UM | Cross-sectional | Urban | Molecular (Xpert®) | 135 | 3 | 2 | 66.7 | 0.04 | Low |
| Davey, 2019 ^10^ † | South Africa | UM | Cross-sectional | Urban | Molecular (Xpert®) | 107 | 9 | 7 | 77.8 | 1.19 | Low |
| Frohlich, 2007 ^11^ | South Africa | LM | Cross-sectional | Rural | Molecular (PCR) | 48 | 2 | 2 | 100 | 0.23 | Low |
| Frohlich, 2007 ^11^ | South Africa | LM | Cross-sectional | Rural | Molecular (PCR) | 226 | 15 | 15 | 100 | 2.52 | Low |
| Ghebremichael, 2014 ^12^ | Tanzania | L | Cross-sectional | Urban | Molecular (PCR) | 1440 | 24 | 17 | 70.8 | 1.39 | Low |
| Hazel, 2014 ^14^ | Namibia | UM | Cross-sectional | Rural | Molecular (PCR) | 209 | 150 | 138 | 92.0 | 9.58 | Low |
| Hoffman, 2019 ^15^ | South Africa | UM | Cross-sectional | Rural | Molecular (PCR) | 251 | 39 | 27 | 69.2 | 27.00 | Low |
| Hokororo, 2015 ^16^ | Tanzania | L | Cross-sectional | Rural | Molecular (PCR) | 403 | 27 | 9 | 33.3 | 2.49 | High |
| Kalsom, 2020 ^18^ | Malaysia | UM | Cross-sectional | Urban | Molecular (PCR) | 137 | 0 | 0 | .. | 0.00 | Low |
| Karim, 2021 ^19^ | Morocco | LM | Cross-sectional | Urban | Molecular (PCR) | 809 | 113 | 83 | 73.5 | 22.93 | High |
| Kerubo, 2016 ^20^ | Kenya | L | Cross-sectional | Rural | Molecular (PCR) | 507 | 3 | 2 | 66.7 | 0.16 | Low |
| Lan, 2008 ^21^ | Vietnam | L | Cross-sectional | Rural | Molecular (PCR) | 1012 | 7 | 4 | 57.1 | 2.30 | Low |
| Lowe, 2019 ^23^ | Zimbabwe | L | Cross-sectional | Urban | Molecular (Xpert®) | 385 | 7 | 4 | 57.1 | 3.74 | High |
| Mahafzah, 2008 ^24^ | Jordan | LM | Cross-sectional | Urban | Molecular (PCR) | 1275 | 14 | 4 | 28.6 | 0.31 | High |
| Mbizvo, 2001 ^26^ | Zimbabwe | L | Cross-sectional | Urban | Molecular (LCR) | 393 | 7 | 2 | 28.6 | 0.25 | Low |
| Moodley, 2015 ^27^ | South Africa | UM | RCT | Urban | Molecular (PCR) | 1459 | 93 | 49 | 52.7 | 0.98 | High |
| Mudau, 2018 ^28^ | South Africa | UM | Cross-sectional | Urban | Molecular (Xpert®) | 247 | 17 | 9 | 52.9 | 1.78 | Low |
| Peters, 2014 ^32^ | South Africa | UM | Cross-sectional | Rural | Molecular (PCR) | 589 | 60 | 41 | 68.3 | 2.85 | Low |
| Rocha, 2014 ^33^ | Brazil | UM | Cross-sectional | Urban | Molecular (PCR) | 361 | 5 | 2 | 40.0 | 4.17 | Low |
| Wangnapi, 2015 ^35^ | PNG | LM | RCT | Both | Molecular (PCR) | 362 | 32 | 3 | 9.4 | 0.74 | Low |
| Warr, 2019 ^36^ | Kenya | L | Cohort | Rural | Molecular (PCR) | 1221 | 29 | 22 | 75.9 | 10.53 | Low |
| ***Trichomonas vaginalis*** | | | | | | | | | | | |
| Adeoye, 2007 ^37^ | Nigeria | L | Cross-sectional | Urban | Microscopy | 544 | 18 | 4 | 22.2 | 0.74 | High |
| Badman, 2016 ^1^ | PNG | LM | Cross-sectional | Urban | Molecular (PCR) | 125 | 47 | 32 | 68.1 | 25.60 | Low |
| Bruce, 2009 ^2^ | PNG | L | Cross-sectional | Urban | Molecular (PCR) | 129 | 53 | 12 | 22.6 | 9.30 | Low |
| Bruni, 2019 ^38^ | Brazil | UM | Cross-sectional | Urban | Molecular (PCR) | 499 | 21 | 4 | 19.0 | 0.80 | High |
| Das, 2013 ^9^ | India | LM | Cohort | Urban | Molecular (PCR) | 417 | 85 | 25 | 29.4 | 6.00 | Low |
| Davey, 2019 ^10^ * | South Africa | UM | Cross-sectional | Urban | Molecular (Xpert®) | 135 | 5 | 4 | 80.0 | 2.96 | Low |
| Davey, 2019 ^10^ † | South Africa | UM | Cross-sectional | Urban | Molecular (Xpert®) | 107 | 16 | 12 | 75.0 | 11.21 | Low |
| de Waaij, 2017 ^39^ | South Africa | UM | Cross-sectional | Rural | Molecular (PCR) | 604 | 75 | 38 | 50.7 | 6.29 | High |
| Frohlich, 2007 ^11^ | South Africa | LM | Cross-sectional | Rural | Molecular (PCR) | 48 | 10 | 10 | 100.0 | 20.83 | Low |
| Frohlich, 2007 ^11^ | South Africa | LM | Cross-sectional | Rural | Molecular (PCR) | 226 | 54 | 54 | 100.0 | 23.89 | Low |
| Ghebremichael, 2014 ^12^ | Tanzania | L | Cross-sectional | Urban | Molecular (PCR) | 1440 | 152 | 94 | 61.8 | 6.53 | Low |
| Hoffman, 2019 ^15^ | South Africa | UM | Cross-sectional | Rural | Molecular (PCR) | 251 | 81 | 50 | 61.7 | 19.92 | Low |
| Kalsom, 2020 ^18^ | Malaysia | UM | Cross-sectional | Urban | Molecular (PCR) | 137 | 1 | 1 | 100.0 | 0.73 | Low |
| Kerubo, 2016 ^20^ | Kenya | L | Cross-sectional | Rural | Molecular (PCR) | 507 | 12 | 6 | 50.0 | 1.18 | Low |
| Lan, 2008 ^21^ | Vietnam | L | Cross-sectional | Rural | Microscopy | 1012 | 10 | 3 | 30.0 | 0.30 | Low |
| Lopez-Monteon, 2013 ^40^ | Mexico | UM | Cross-sectional | Urban | Molecular (PCR) | 210 | 48 | 45 | 93.8 | 21.43 | Low |
| Lowe, 2019 ^23^ | Zimbabwe | L | Cross-sectional | Urban | Molecular (Xpert®) | 385 | 31 | 19 | 61.3 | 4.94 | High |
| Mabaso, 2020 ^41^ | South Africa | UM | Cross-sectional | Urban | Molecular (PCR) | 362 | 47 | 28 | 59.6 | 7.73 | High |
| Mahafzah, 2008 ^24^ | Jordan | LM | Cross-sectional | Urban | InPouch® | 1275 | 9 | 1 | 11.1 | 0.08 | High |
| Mbizvo, 2001 ^26^ | Zimbabwe | L | Cross-sectional | Urban | Microscopy | 393 | 60 | 40 | 66.7 | 10.18 | Low |
| Miranda, 2014 ^42^ | Brazil | UM | Cross-sectional | Both | Molecular (PCR) | 299 | 23 | 16 | 69.6 | 5.35 | High |
| Moodley, 2015 ^27^ | South Africa | UM | RCT | Urban | Molecular (PCR) | 1459 | 223 | 118 | 52.9 | 8.09 | High |
| Mucci, 2016 ^43^ | Argentina | UM | Cross-sectional | Urban | Microscopy | 210 | 3 | 0 | 0.0 | 0.00 | High |
| Mudau, 2018 ^28^ | South Africa | UM | Cross-sectional | Urban | Molecular (PCR) | 247 | 59 | 43 | 72.9 | 17.41 | Low |
| Paul, 2012 ^44^ | India | LM | Cross-sectional | Urban | Molecular (PCR) | 198 | 10 | 7 | 70.0 | 3.54 | High |
| Rassjö, 2006 ^45^ | Uganda | L | Cross-sectional | Urban | Molecular (PCR) | 199 | 16 | 7 | 43.8 | 3.52 | Low |
| Rocha, 2014 ^33^ | Brazil | UM | Cross-sectional | Urban | Molecular (PCR) | 361 | 46 | 21 | 45.7 | 5.82 | Low |
| Shehabi, 2009 ^46^ | Jordan | LM | Cross-sectional | Urban | Molecular (PCR) | 182 | 1 | 0 | 0.0 | 0.00 | High |
| Tann, 2006 ^47^ | Uganda | L | Cross-sectional | Urban | InPouch® | 250 | 244 | 13 | 5.3 | 5.20 | Low |
| Tavakoli Oliaee, 2017 ^48^ | Iran | UM | Cross-sectional | Urban | Molecular (PCR) | 150 | 24 | 2 | 8.3 | 1.33 | High |
| Wangnapi, 2015 ^35^ | PNG | LM | RCT | Both | Molecular (PCR) | 362 | 73 | 15 | 20.5 | 4.14 | Low |

| L: Low-income country LM: Lower middle-income country UM: Upper middle-income country PNG: Papua New Guinea RCT: Randomised controlled trial | * Pregnant and without HIV † Pregnant and with HIV ‡ Obstetric ward § Gynaecology ward ¶ Infertility ward |
| --- | --- |
