# Supplementary material for: Asymptomatic infections with Chlamydia trachomatis, Neisseria gonorrhoeae, and Trichomonas vaginalis among women in low- and middle-income countries: A systematic review and meta-analysis
Source: PLOS Glob Public Health. 2024 May 23;4(5):e0003226. doi: 10.1371/journal.pgph.0003226 (PMC11115196; doi:10.1371/journal.pgph.0003226)
Supplement: S3 Table — (DOCX) [file pgph.0003226.s006.docx]

**S3 Table: Proportion and prevalence of asymptomatic CT infections: number of studies, number of participants, and I² included**

|  |  | **Number of asymptomatic** | **Number of positive** | **Study population** | **Number of data points** | **Number of countries** | **Pooled proportion estimates in % [95% CI]** | **Heterogeneity I² for proportion** | **P-value for subgroup analysis** | **Pooled prevalence (per 100 women) estimates [95% CI]** | **Heterogeneity I² for prevalence** | **P-value for subgroup analysis** |
| --- | --- | --- | --- | --- | --- | --- | --- | --- | --- | --- | --- | --- |
| **Overall** | |  |  |  |  |  |  |  |  |  |  |  |
|  | Excluding populations with an increased risk of STI* | 910 | 1718 | 26 268 | 33 | 17 | 60.7 [50.4; 70.5] | 93.2% | .. | 4.70 [3.39; 6.20] | 96.1% | .. |
|  | Including populations with an increased risk of STI | 1361 | 2462 | 28 897 | 41 | 18 | 58.8 [50.4; 67.0] | 93.2% | .. | 6.05 [4.26; 8.12] | 97.7% | .. |
| **Continent*** | |  |  |  |  |  |  |  |  |  |  |  |
|  | Africa | 470 | 751 | 12 368 | 14 | 6 | 68.6 [56.4; 79.8] | 87.8% | 0.048 | 4.99 [2.70; 7.90] | 97.3% | 0.068 |
|  | Asia | 370 | 731 | 10 563 | 12 | 6 | 66.2 [47.2; 83.0] | 94.6% |  | 5.55 [3.40; 8.19] | 95.5% |  |
|  | Latin America | 43 | 172 | 2 850 | 5 | 4 | 30.9 [11.2; 54.8] | 89.4% |  | 1.89 [0.56; 3.91] | 89.6% |  |
|  | Oceania | 27 | 64 | 487 | 2 | 1 | 47.4 [2.7; 95.0] | 95.0% |  | 7.22 [0.00; 24.73] | 95.7% |  |
| **Country income level*** | |  |  |  |  |  |  |  |  |  |  |  |
|  | Low-Income | 251 | 442 | 11 381 | 11 | 6 | 55.5 [37.3; 73.1] | 91.8% | 0.482 | 3.55 [1.87; 5.70] | 96.0% | 0.184 |
|  | Middle-Income | 659 | 1 276 | 14 887 | 22 | 11 | 63.2 [50.5; 75.1] | 94.0% |  | 5.34 [3.59; 7.39] | 95.6% |  |
| **Setting*** | |  |  |  |  |  |  |  |  |  |  |  |
|  | Rural | 311 | 477 | 9 150 | 10 | 5 | 71.6 [55.6; 85.3] | 91.0% | 0.106 | 5.36 [3.11; 8.15] | 95.2% | 0.746 |
|  | Urban | 539 | 1 124 | 15 276 | 20 | 14 | 55.6 [43.8; 67.1] | 90.9% |  | 4.83 [2.90; 7.19] | 97.0% |  |
| **Study year*** | |  |  |  |  |  |  |  |  |  |  |  |
|  | 1998 - 2011 | 478 | 789 | 13 987 | 17 | 12 | 61.8 [48.1; 74.7] | 90.8% | 0.727 | 4.41 [2.37; 6.99] | 97.4% | 0.782 |
|  | 2012 - 2022 | 403 | 895 | 12 181 | 15 | 9 | 57.7 [42.1; 72.7] | 94.2% |  | 4.09 [2.88; 5.49] | 90.6% |  |
| **Number of symptoms assessed*** | | |  |  |  |  |  |  |  |  |  |  |
|  | Between 1 and 4 | 398 | 734 | 6 917 | 13 | 10 | 57.4 [41.6; 72.4] | 93.6% | 0.345 | 6.53 [3.46; 10.45] | 96.9% | 0.267 |
|  | Five and more | 265 | 420 | 8 137 | 15 | 8 | 69.0 [51.7; 84.2] | 90.9% |  | 4.29 [2.48; 6.53] | 94.6% |  |
| **Key population**** | |  |  |  |  |  |  |  |  |  |  |  |
|  | Pregnant women | 377 | 708 | 5 749 | 12 | 7 | 55.3 [40.6; 69.6] | 92.0% | .. | 7.50 [4.31; 11.44] | 95.7% | .. |
|  | Female sex workers | 371 | 624 | 1 890 | 5 | 4 | 45.5 [24.8; 67.0] | 96.0% | .. | 13.63 [2.76; 30.72] | 98.8% | .. |
|  | Adolescents | 109 | 172 | 5 596 | 3 | 2 | 57.4 [17.9; 92.2] | 95.4% | .. | 1.89 [1.55; 2.27] | 0% | .. |
|  | Women with HIV | 80 | 120 | 739 | 3 | 2 | 64.7 [48.3; 79.7] | 47.7% | .. | 10.11 [0.13; 31.39] | 98.0% | .. |
|  | Infertile | 81 | 111 | 755 | 4 | 3 | 74.7 [59.9; 87.2] | 58.2% | .. | 11.76 [4.33; 22.07] | 92.0% | .. |

* Excludes populations with an increased risk of STI (FSW, women with HIV, and women attending an STI clinic)
** "Pregnant women" and "Women with HIV" are not mutually exclusive
