# Supplementary material for: Asymptomatic infections with Chlamydia trachomatis, Neisseria gonorrhoeae, and Trichomonas vaginalis among women in low- and middle-income countries: A systematic review and meta-analysis
Source: PLOS Glob Public Health. 2024 May 23;4(5):e0003226. doi: 10.1371/journal.pgph.0003226 (PMC11115196; doi:10.1371/journal.pgph.0003226)
Supplement: S4 Table — (DOCX) [file pgph.0003226.s007.docx]

**S4 Table: Proportion and prevalence of asymptomatic NG infections: number of studies, number of participants, and I²**

|  |  | **Number of asymptomatic** | **Number of positive** | **Study population** | **Number of data points** | **Number of countries** | **Pooled proportion estimates in % [95% CI]** | **Heterogeneity I² for proportion** | **P-value for subgroup analysis** | **Pooled prevalence (per 100 women) estimates [95% CI]** | **Heterogeneity I² for prevalence** | **P-value for subgroup analysis** |
| --- | --- | --- | --- | --- | --- | --- | --- | --- | --- | --- | --- | --- |
| **Overall** | |  |  |  |  |  |  |  |  |  |  |  |
|  | Excluding populations with an increased risk of STI* | 423 | 685 | 16 950 | 21 | 13 | 53.3 [37.1; 69.1] | 92.3% | .. | 3.11 [1.34; 5.54] | 98.2% | .. |
|  | Including populations with an increased risk of STI | 606 | 1 000 | 18 740 | 27 | 14 | 52.2 [38.5; 65.6] | 92.8% | .. | 3.70 [1.79; 6.23] | 98.3% | .. |
| **Continent*** | |  |  |  |  |  |  |  |  |  |  |  |
|  | Africa | 394 | 565 | 7 681 | 13 | 6 | 67.2 [51.0; 81.9] | 90.1% | 0.019 | 5.67 [2.15; 10.66] | 98.4% | <0.001 |
|  | Asia | 15 | 64 | 7 236 | 4 | 4 | 27.7 [9.0; 50.9] | 59.7% |  | 0.13 [0.03; 0.28] | 12.6% |  |
|  | Latin America | 2 | 10 | 1 546 | 2 | 2 | 14.0 [0.0; 67.5] | 62.2% |  | 0.12 [0.00; 1.16] | 80.6% |  |
|  | Oceania | 12 | 46 | 487 | 2 | 1 | 32.7 [0.0; 88.3] | 92.8% |  | 3.12 [0.00; 12.25] | 91.7% |  |
| **Country income level*** | |  |  |  |  |  |  |  |  |  |  |  |
|  | Low income | 41 | 102 | 6 157 | 7 | 5 | 37.7 [18.4; 58.8] | 69.2% | 0.116 | 0.57 [0.17; 1.17] | 82.9% | 0.002 |
|  | Middle income | 382 | 583 | 10 793 | 14 | 8 | 60.9 [41.1; 79.2] | 93.9% |  | 5.21 [1.77; 10.22] | 98.7% |  |
| **Setting*** | |  |  |  |  |  |  |  |  |  |  |  |
|  | Rural | 245 | 332 | 4 464 | 9 | 5 | 70.4 [46.3; 90.3] | 91.9% | 0.093 | 6.80 [1.39; 15.60] | 98.8% | 0.059 |
|  | Urban | 175 | 321 | 12 124 | 11 | 10 | 44.4 [26.5; 62.9] | 85.8% |  | 1.33 [0.29; 3.02] | 97.0% |  |
| **Study year*** | |  |  |  |  |  |  |  |  |  |  |  |
|  | 1998 - 2011 | 277 | 414 | 8 552 | 12 | 9 | 55.9 [32.9; 77.8] | 93.2% | 0.675 | 3.72 [0.98; 7.99] | 98.6% | 0.554 |
|  | 2012 - 2022 | 146 | 271 | 8 398 | 9 | 7 | 48.9 [26.9; 71.1] | 89.4% |  | 2.42 [0.47; 5.63] | 97.5% |  |
| **Number of symptoms assessed*** | | |  |  |  |  |  |  |  |  |  |  |
|  | Between 1 and 4 | 240 | 376 | 5 548 | 8 | 7 | 40.6 [13.8; 70.4] | 96.2% | 0.079 | 4.39 [0.46; 11.76] | 99.0% | 0.806 |
|  | Five and more | 170 | 247 | 4 954 | 10 | 6 | 71.8 [56.6; 85.2] | 70.1% |  | 3.50 [1.27; 6.68] | 95.6% |  |
| **Key population**** | |  |  |  |  |  |  |  |  |  |  |  |
|  | Pregnant women | 97 | 226 | 4 106 | 9 | 4 | 45.7 [27.9; 63.9] | 79.4% | .. | 2.60 [1.27; 4.32] | 85.1% | .. |
|  | Female sex workers | 163 | 282 | 1 051 | 3 | 3 | 38.1 [3.1; 82.9] | 97.9% | .. | 9.49 [0.04; 30.73] | 98.6% | .. |
|  | Adolescents | 11 | 30 | 910 | 2 | 2 | 35.9 [15.0; 59.2] | 7.2% | .. | 1.12 [0.01; 3.59] | 84.3% | .. |
|  | Women with HIV | 20 | 33 | 739 | 3 | 2 | 61.0 [42.7; 78.0] | 0% | .. | 3.09 [0.68; 6.96] | 80.8% | .. |
|  | Infertile | 0 | 0 | 137 | 1 | 1 | .. | .. | .. | 0.00 [0.07; 3.40] | .. | .. |

* Excludes populations with an increased risk of STI (FSW, women with HIV, and women attending an STI clinic)
** "Pregnant women" and "Women with HIV" are not mutually exclusive
