# Supplementary material for: Asymptomatic infections with Chlamydia trachomatis, Neisseria gonorrhoeae, and Trichomonas vaginalis among women in low- and middle-income countries: A systematic review and meta-analysis
Source: PLOS Glob Public Health. 2024 May 23;4(5):e0003226. doi: 10.1371/journal.pgph.0003226 (PMC11115196; doi:10.1371/journal.pgph.0003226)
Supplement: S5 Table — (DOCX) [file pgph.0003226.s008.docx]

**S5 Table: Proportion and prevalence of asymptomatic TV infections: number of studies, number of participants, and I²**

|  |  | **Number of asymptomatic** | **Number of positive** | **Study population** | **Number of data points** | **Number of countries** | **Pooled proportion estimates in % [95% CI]** | **Heterogeneity I² for proportion** | **P-value for subgroup analysis** | **Pooled prevalence (per 100 women) estimates [95% CI]** | **Heterogeneity I² for prevalence** | **P-value for subgroup analysis** |
| --- | --- | --- | --- | --- | --- | --- | --- | --- | --- | --- | --- | --- |
| **Overall** | |  |  |  |  |  |  |  |  |  |  |  |
|  | Excluding populations with an increased risk of STI* | 642 | 1 111 | 10 824 | 23 | 13 | 56.9 [44.6; 68.9] | 91.4% | .. | 5.98 [3.46; 9.12] | 97.3% | .. |
|  | Including populations with an increased risk of STI | 761 | 1 394 | 12 684 | 31 | 15 | 53.6 [43.0; 64.1] | 91.3% | .. | 5.84 [3.75; 8.34] | 96.6% | .. |
| **Continent*** | |  |  |  |  |  |  |  |  |  |  |  |
|  | Africa | 504 | 829 | 6 152 | 12 | 6 | 64.9 [52.2; 76.7] | 91.5% | 0.039 | 9.06 [5.71; 13.07] | 95.7% | <0.001 |
|  | Asia | 5 | 21 | 2 606 | 4 | 3 | 17.3 [0.0; 49.5] | 15.3% |  | 0.08 [0.00; 0.31] | 7.2% |  |
|  | Latin America | 86 | 141 | 1 579 | 5 | 3 | 50.1 [16.4; 83.8] | 93.1% |  | 4.54 [0.52; 11.9] | 96.6% |  |
|  | Oceania | 47 | 120 | 487 | 2 | 1 | 43.3 [4.8; 87.5] | 96.4% |  | 12.77 [0.06; 40.12] | 97.5% |  |
| **Country income level*** | |  |  |  |  |  |  |  |  |  |  |  |
|  | Low income | 160 | 295 | 4 136 | 6 | 6 | 45.4 [29.7; 61.6] | 92.8% | 0.174 | 3.12 [0.73; 7.02] | 96.9% | 0.108 |
|  | Middle income | 482 | 816 | 6 688 | 17 | 7 | 61.7 [45.4; 77.1] | 81.7% |  | 7.28 [3.72; 11.88] | 97.4% |  |
| **Setting*** | |  |  |  |  |  |  |  |  |  |  |  |
|  | Rural | 198 | 280 | 2 619 | 6 | 3 | 75.0 [49.8; 94.0] | 93.2% | 0.090 | 10.27 [2.41 22.46] | 98.5% | 0.252 |
|  | Urban | 413 | 735 | 7 544 | 15 | 11 | 58.9 [46.5; 70.8] | 86.0% |  | 4.81 [2.25; 8.21] | 97.0% |  |
| **Study year*** | |  |  |  |  |  |  |  |  |  |  |  |
|  | 1998 - 2011 | 460 | 817 | 7 872 | 13 | 8 | 57.3 [41.0; 73.0] | 93.5% | 0.797 | 5.94 [2.86; 10.00] | 97.6% | 0.759 |
|  | 2012 - 2022 | 178 | 276 | 2 408 | 9 | 7 | 61.1 [41.3; 79.5] | 85.2% |  | 6.99 [2.09; 14.30] | 97.0% |  |
| **Number of symptoms assessed*** | | |  |  |  |  |  |  |  |  |  |  |
|  | Between 1 and 4 | 355 | 650 | 4 763 | 11 | 9 | 55.1 [37.7; 71.9] | 91.1% | 0.487 | 5.68 [2.97; 9.17] | 95.0% | 0.518 |
|  | Five and more | 265 | 406 | 4 425 | 10 | 7 | 63.6 [43.3; 81.9] | 91.9% |  | 7.66 [2.94; 14.24] | 97.7% |  |
| **Key population**** | |  |  |  |  |  |  |  |  |  |  |  |
|  | Pregnant women | 291 | 549 | 3 598 | 11 | 5 | 58.8 [43.9; 73.0] | 88.1% | .. | 7.96 [4.73; 11.91] | 92.5% | .. |
|  | Female sex workers | 37 | 138 | 546 | 2 | 2 | 26.7 [19.6; 34.5] | 0% | .. | 7.02 [4.16; 10.52] | 41.3% | .. |
|  | Adolescents | 13 | 28 | 706 | 2 | 2 | 46.4 [27.6; 65.7] | 0% | .. | 2.05 [0.33; 4.98] | 73.7% | .. |
|  | Women with HIV | 73 | 105 | 965 | 4 | 3 | 70.5 [60.7; 79.6] | 0% | .. | 6.42 [1.89; 13.23] | 92.0% | .. |
|  | Infertile | 1 | 1 | 137 | 1 | 1 | 100 [5.5; 89.2] | .. | .. | 0.73 [0.04; 4.60] | .. | .. |

* Excludes populations with an increased risk of STI (FSW, women with HIV, and women attending an STI clinic)
** "Pregnant women" and "Women with HIV" are not mutually exclusive
