# Supplementary material for: Asymptomatic infections with Chlamydia trachomatis, Neisseria gonorrhoeae, and Trichomonas vaginalis among women in low- and middle-income countries: A systematic review and meta-analysis
Source: PLOS Glob Public Health. 2024 May 23;4(5):e0003226. doi: 10.1371/journal.pgph.0003226 (PMC11115196; doi:10.1371/journal.pgph.0003226)
Supplement: S6 Table — (DOCX) [file pgph.0003226.s009.docx]

**S6 Table: Proportion and prevalence of asymptomatic CT, NG, and TV by country (data used for the map)**

|  |  | Asymptomatic CT | | | | Asymptomatic NG | | | | | Asymptomatic TV | | | | |
| --- | --- | --- | --- | --- | --- | --- | --- | --- | --- | --- | --- | --- | --- | --- | --- |
| **Country** | **N** | **Proportion** | **95% CI** | **Prevalence** | **95% CI** | **N** | **Proportion** | **95% CI** | **Prevalence** | **95% CI** | **N** | **Proportion** | **95% CI** | **Prevalence** | **95% CI** |
| Argentina | .. | .. | .. | .. | .. | .. | .. | .. | .. | .. | 1 | 0.0 | [0.0; 50.0] | 0.00 | [0.00; 0.82] |
| Brazil | 2 | 38.8 | [10.5; 71.8] | 2.51 | [0.96; 4.73] | 1 | 40.0 | [1.9; 86.2] | 0.55 | [0.01; 1.66] | 3 | 44.5 | [19.8; 70.5] | 3.50 | [0.67; 8.28] |
| China | 5 | 70.6 | [34.5; 96.4] | 3.57 | [2.78; 4.47] | 1 | 16.3 | [6.5; 29.0] | 0.15 | [0.05; 0.28] | .. | .. | .. | .. | .. |
| India | 3 | 76.0 | [61.5; 88.0] | 15.98 | [7.72; 26.44] | .. | .. | .. | .. | .. | .. | .. | .. | .. | .. |
| Iran | 1 | 35.3 | [14.0; 59.8] | 7.50 | [2.57; 14.46] | .. | .. | .. | .. | .. | .. | .. | .. | .. | .. |
| Jordan | 1 | 14.3 | [0.00; 51.7] | 0.08 | [0.00; 0.34] | 1 | 28.6 | [7.4; 55.4] | 0.31 | [0.07; 0.71] | 2 | 2.5 | [0.0; 36.0] | 0.01 | [0.00; 0.22] |
| Kenya | 2 | 52.9 | [28.5; 76.7] | 2.13 | [1.49; 2.87] | 2 | 33.2 | [ 2.4; 73.4] | 0.51 | [0.21; 0.92] | 1 | 50.0 | [21.6; 78.4] | 1.18 | [0.39; 2.35] |
| Malaysia | 1 | 86.7 | [63.8; 99.7] | 9.49 | [5.08; 15.03] | .. | ..* | .. | 0.00 | [0.00; 1.25] | 1 | 100 | [0.0; 100] | 0.73 | [0.00; 3.11] |
| Mexico | 1 | 73.3 | [47.7; 93.2] | 4.78 | [2.35; 7.97] | .. | .. | .. |  |  | 1 | 93.8 | [84.7; 99.2] | 21.43 | [16.12; 27.25] |
| Morocco | 1 | 53.8 | [26.1; 80.5] | 0.87 | [0.32; 1.65] | 1 | 73.5 | [64.9; 81.2] | 10.29 | [8.28; 12.48] | .. | .. | .. | .. | .. |
| Namibia | 1 | 95.8 | [83.0; 100] | 11.11 | [7.16; 15.79] | 1 | 92.0 | [87.1; 95.9] | 66.7 | [60.08; 72.94] | .. | .. | .. | .. | .. |
| Nicaragua | 1 | 7.5 | [1.0; 18.1] | 0.25 | [0.03; 0.64] | 1 | 0.0 | [0.0; 31.7] | 0.00 | [0.00; 0.15] | .. | .. | .. | .. | .. |
| Nigeria | .. | .. | .. | .. | .. | .. | .. | .. | .. | .. | 1 | 22.2 | [5.5; 44.8] | 0.74 | [0.16; 1.67] |
| Peru | 1 | 13.3 | [5.7; 23.3] | 1.33 | [0.55; 2.43] | .. | .. | .. | .. | .. | .. | .. | .. | .. | .. |
| PNG | 2 | 47.4 | [2.7; 95.1] | 7.22 | [0.00; 24.73] | 2 | 32.7 | [0.0; 88.3] | 3.12 | [0.00; 12.25] | 2 | 43.3 | [4.8; 87.5] | 12.77 | [0.06; 40.12] |
| South Africa | 6 | 76.2 | [61.6; 88.4] | 10.72 | [9.47; 12.03] | 6 | 76.8 | [57.7; 92.2] | 5.29 | [2.95; 8.21] | 7 | 76.6 | [59.0; 90.6] | 13.97 | [9.50; 19.12] |
| Tanzania | 3 | 51.8 | [4.9; 96.9] | 1.19 | [0.06; 3.58] | 2 | 52.0 | [17.2; 85.9] | 1.53 | [0.66; 2.74] | 1 | 61.8 | [54.0; 69.4] | 6.53 | [5.31; 7.86] |
| Uganda | .. | .. | .. | .. | .. | .. | .. | .. | .. | .. | 1 | 30.2 | [17.3; 44.9] | 5.33 | [2.82; 8.54] |
| Vietnam | 1 | 55.8 | [40.7; 70.4] | 2.37 | [1.52; 3.41] | 1 | 57.1 | [18.6; 91.9] | 0.40 | [0.08; 0.90] | 1 | 30.0 | [5.0; 62.5] | 0.30 | [0.04; 0.75] |

N represents the number of point estimates
PNG: Papua New-Guinea

* Only one study represented Malaysia and no women were tested positive for NG in this study
