# Supplementary material for: Asymptomatic infections with Chlamydia trachomatis, Neisseria gonorrhoeae, and Trichomonas vaginalis among women in low- and middle-income countries: A systematic review and meta-analysis
Source: PLOS Glob Public Health. 2024 May 23;4(5):e0003226. doi: 10.1371/journal.pgph.0003226 (PMC11115196; doi:10.1371/journal.pgph.0003226)
Supplement: S7 Table — (DOCX) [file pgph.0003226.s010.docx]

**S7 Table: Sensitivity analysis – Proportion and prevalence estimates of asymptomatic CT infections excluding studies with a high risk of bias**

|  |  | **Number of asymptomatic** | **Number of positive** | **Study population** | **Number of data points** | **Number of countries** | **Pooled proportion estimates in % [95% CI]** | **Heterogeneity I² for proportion** | **P-value for subgroup analysis** | **Pooled prevalence (per 100 women) estimates [95% CI]** | **Heterogeneity I² for prevalence** | **P-value for subgroup analysis** |
| --- | --- | --- | --- | --- | --- | --- | --- | --- | --- | --- | --- | --- |
| **Overall** | |  |  |  |  |  |  |  |  |  |  |  |
|  | Excluding populations with an increased risk of STI* | 510 | 852 | 16 664 | 23 | 12 | 64.2 [50.1; 77.3] | 93.2% | .. | 4.23 [2.85; 5.85] | 95.1% | .. |
|  | Including populations with an increased risk of STI | 995 | 1588 | 18 908 | 30 | 14 | 60.3 [49.8; 70.4] | 93.4% | .. | 6.29 [3.99; 9.05] | 97.9% | .. |
| **Continent*** | |  |  |  |  |  |  |  |  |  |  |  |
|  | Africa | 340 | 433 | 9 699 | 11 | 5 | 75.9 [63.0; 87.1] | 82.9% | 0.001 | 5.36 [2.76; 8.70] | 96.8% | 0.044 |
|  | Asia | 147 | 198 | 3 858 | 6 | 3 | 77.0 [53.9; 94.2] | 91.1% |  | 3.93 [2.78; 5.27] | 71.5% |  |
|  | Latin America | 32 | 157 | 2 620 | 4 | 3 | 22.3 [7.2; 42.1] | 85.3% |  | 1.42 [0.35; 3.18] | 88.4% |  |
|  | Oceania | 27 | 64 | 487 | 2 | 1 | 47.4 [2.7; 95.0] | 95.0% |  | 7.22 [0.00; 24.73] | 95.7% |  |
| **Country income level*** | |  |  |  |  |  |  |  |  |  |  |  |
|  | Low income | 162 | 291 | 10 440 | 7 | 5 | 50.1 [25.4; 74.8] | 92.6% | 0.194 | 1.29 [0.53; 2.34] | 92.9% | 0.001 |
|  | Middle income | 348 | 561 | 6 224 | 16 | 7 | 69.7 [52.2; 84.8] | 93.9% |  | 6.24 [4.25; 8.57] | 91.3% |  |
| **Setting*** | |  |  |  |  |  |  |  |  |  |  |  |
|  | Rural | 300 | 431 | 8 747 | 9 | 5 | 76.4 [62.8; 87.9] | 86.7% | 0.082 | 5.74 [3.20; 8.92] | 95.7% | 0.243 |
|  | Urban | 150 | 304 | 6 075 | 11 | 9 | 54.7 [32.9; 75.6] | 92.0% |  | 3.71 [1.69; 6.43] | 95.4% |  |
| **Study year*** | |  |  |  |  |  |  |  |  |  |  |  |
|  | 1998 - 2011 | 263 | 420 | 10 505 | 11 | 8 | 65.4 [43.5; 84.7] | 93.6% | 0.925 | 3.49 [1.64; 5.93] | 96.4% | 0.343 |
|  | 2012 - 2022 | 247 | 432 | 6 159 | 12 | 7 | 63.3 [43.6; 81.0] | 93.8% |  | 4.92 [3.19; 7.00] | 91.0% |  |
| **Number of symptoms assessed*** | | |  |  |  |  |  |  |  |  |  |  |
|  | Between 1 and 4 | 161 | 355 | 6 917 | 8 | 8 | 48.2 [24.0; 72.8] | 95.3% | 0.104 | 3.92 [1.43; 7.49] | 96.3% | 0.613 |
|  | Five and more | 247 | 361 | 8 137 | 13 | 7 | 73.9 [56.8; 88.3] | 89.4% |  | 4.87 [2.69; 7.61] | 95.1% |  |
| **Key population**** | |  |  |  |  |  |  |  |  |  |  |  |
|  | Pregnant women | 199 | 379 | 3 713 | 9 | 5 | 56.7 [36.4; 76.2] | 92.9% | .. | 7.59 [3.65; 12.7] | 95.7% | .. |
|  | Female sex workers | 371 | 624 | 1 890 | 5 | 4 | 45.5 [24.8; 67.0] | 96.0% | .. | 13.63 [2.76; 30.72] | 98.8% | .. |
|  | Adolescents | 98 | 126 | 5 193 | 2 | 2 | 78.6 [70.6; 85.7] | 0% | .. | 1.85 [1.50; 2.24] | 0% | .. |
|  | Women with HIV | 74 | 112 | 354 | 2 | 1 | 61.4 [38.7; 81.8] | 72.8% | .. | 17.1 [4.27; 35.8] | 93.0% | .. |
|  | Infertile | 22 | 30 | 391 | 2 | 2 | 74.3 [45.4; 95.6] | 60.6% | .. | 6.01 [1.46; 13.14] | 81.8% | .. |

* Excludes populations with an increased risk of STI (FSW, women with HIV, and women attending an STI clinic)
** "Pregnant women" and "Women with HIV" are not mutually exclusive
