# Supplementary material for: Asymptomatic infections with Chlamydia trachomatis, Neisseria gonorrhoeae, and Trichomonas vaginalis among women in low- and middle-income countries: A systematic review and meta-analysis
Source: PLOS Glob Public Health. 2024 May 23;4(5):e0003226. doi: 10.1371/journal.pgph.0003226 (PMC11115196; doi:10.1371/journal.pgph.0003226)
Supplement: S8 Table — (DOCX) [file pgph.0003226.s011.docx]

**S8 Table: Sensitivity analysis – Proportion and prevalence estimates of asymptomatic NG infections excluding studies with a high risk of bias**

|  |  | **Number of asymptomatic** | **Number of positive** | **Study population** | **Number of data points** | **Number of countries** | **Pooled proportion estimates in % [95% CI]** | **Heterogeneity I² for proportion** | **P-value for subgroup analysis** | **Pooled prevalence (per 100 women) estimates [95% CI]** | **Heterogeneity I² for prevalence** | **P-value for subgroup analysis** |
| --- | --- | --- | --- | --- | --- | --- | --- | --- | --- | --- | --- | --- |
| **Overall** | |  |  |  |  |  |  |  |  |  |  |  |
|  | Excluding populations with an increased risk of STI* | 271 | 395 | 8 195 | 16 | 10 | 58.4 [36.7; 78.7] | 91.6% | .. | 3.46 [1.02; 7,14] | 98.1% | .. |
|  | Including populations with an increased risk of STI | 450 | 703 | 9 600 | 21 | 12 | 55.3 [38.0; 72.1] | 93.1% | .. | 4.31 [1.61; 8.14] | 98.3% | .. |
| **Continent*** | |  |  |  |  |  |  |  |  |  |  |  |
|  | Africa | 253 | 332 | 5 013 | 10 | 5 | 72.3 [50.9; 90.1] | 89.1% | 0.019 | 5.98 [1.35; 13.38] | 98.6% | 0.016 |
|  | Asia | 4 | 7 | 1 149 | 2 | 2 | 57.7 [18.6; 91.9] | .. |  | 0.23 [0.00; 0.66] | 0% |  |
|  | Latin America | 2 | 10 | 1 546 | 2 | 2 | 14.0 [0.0; 67.5] | 62.2% |  | 0.12 [0.00; 1.16] | 80.6% |  |
|  | Oceania | 12 | 46 | 487 | 2 | 1 | 32.7 [0.0; 88.3] | 92.8% |  | 3.12 [0.00; 12.25] | 91.7% |  |
| **Country income level*** | |  |  |  |  |  |  |  |  |  |  |  |
|  | Low income | 32 | 75 | 5 754 | 6 | 5 | 38.6 [14.3; 65.7] | 73.6% | 0.099 | 0.42 [0.10; 0.92] | 79.6% | 0.013 |
|  | Middle income | 239 | 320 | 2 441 | 10 | 5 | 70.6 [43.7; 92.4] | 93.0% |  | 6.80 [1.00; 16.7] | 98.3% |  |
| **Setting*** | |  |  |  |  |  |  |  |  |  |  |  |
|  | Rural | 236 | 305 | 4 061 | 8 | 4 | 75.6 [51.7; 94.1] | 90.7% | 0.092 | 7.55 [1.29; 18.06] | 99.0% | 0.032 |
|  | Urban | 32 | 58 | 3 772 | 7 | 7 | 45.8 [21.2; 71.3] | 63.4% |  | 0.80 [0.08; 2.06] | 87.4% |  |
| **Study year*** | |  |  |  |  |  |  |  |  |  |  |  |
|  | 1998 - 2011 | 224 | 307 | 5 819 | 10 | 8 | 59.2 [30.8; 85.1] | 93.4% | 0.838 | 4.35 [0.71; 10.60] | 98.7% | 0.433 |
|  | 2012 - 2022 | 47 | 88 | 2 376 | 6 | 4 | 55.3 [29.6; 80.3] | 76.4% |  | 2.16 [0.23; 5.64] | 93.5% |  |
| **Number of symptoms assessed*** | | |  |  |  |  |  |  |  |  |  |  |
|  | Between 1 and 4 | 191 | 283 | 4 090 | 7 | 7 | 37.6 [6.2; 75.6] | 96.7% | 0.067 | 4.56 [0.09; 14.43] | 99.1% | 0.686 |
|  | Five and more | 78 | 107 | 3 744 | 8 | 5 | 78.3 [62.2; 91.5] | 49.3% |  | 2.94 [1.00; 5.74] | 92.8% |  |
| **Key population**** | |  |  |  |  |  |  |  |  |  |  |  |
|  | Pregnant women | 39 | 106 | 2 245 | 7 | 3 | 48.9 [22.9; 75.3] | 81.3% | .. | 2.65 [0.89; 5.18] | 84.7% | .. |
|  | Female sex workers | 163 | 282 | 1 051 | 3 | 3 | 38.1 [3.1; 82.9] | 97.9% | .. | 9.49 [0.04; 30.73] | 98.6% | .. |
|  | Adolescents | 2 | 30 | 507 | 1 | 1 | 66.7 [12.5; 98.2] | .. | .. | 0.40 [0.07; 1.58] | .. | .. |
|  | Women with HIV | 16 | 26 | 354 | 2 | 1 | 62.9 [38.7; 84.4] | 27.5% | .. | 4.56 [2.13; 7.79] | 31.5% | .. |
|  | Infertile | 0 | 0 | 137 | 1 | 1 | .. | .. | .. | 0.00 [0.07; 3.40] | .. | .. |

* Excludes populations with an increased risk of STI (FSW, women with HIV, and women attending an STI clinic)
** "Pregnant women" and "Women with HIV" are not mutually exclusive
