# Supplementary material for: Asymptomatic infections with Chlamydia trachomatis, Neisseria gonorrhoeae, and Trichomonas vaginalis among women in low- and middle-income countries: A systematic review and meta-analysis
Source: PLOS Glob Public Health. 2024 May 23;4(5):e0003226. doi: 10.1371/journal.pgph.0003226 (PMC11115196; doi:10.1371/journal.pgph.0003226)
Supplement: S9 Table — (DOCX) [file pgph.0003226.s012.docx]

**S9 Table: Sensitivity analysis – Proportion and prevalence estimates of asymptomatic TV infections excluding studies with a high risk of bias**

|  |  | **Number of asymptomatic** | **Number of positive** | **Study population** | **Number of data points** | **Number of countries** | **Pooled prevalence (per 100 women) estimates [95% CI]** | **Heterogeneity I² for prevalence** | **P-value for subgroup analysis** | **Pooled proportion estimates in % [95% CI]** | **Heterogeneity I² for proportion** | **P-value for subgroup analysis** |
| --- | --- | --- | --- | --- | --- | --- | --- | --- | --- | --- | --- | --- |
| **Overall** | |  |  |  |  |  |  |  |  |  |  |  |
|  | Excluding populations with an increased risk of STI* | 396 | 653 | 5 419 | 14 | 10 | 9.02 [4.94; 14.13] | 96.9% | .. | 66.9 [49.4; 82.5] | 93.6% | .. |
|  | Including populations with an increased risk of STI | 487 | 871 | 6 546 | 19 | 11 | 8.49 [5.23; 12.43] | 96.1% | .. | 61.5 [46.7; 75.5] | 93.4% | .. |
| **Continent*** | |  |  |  |  |  |  |  |  |  |  |  |
|  | Africa | 279 | 428 | 3 212 | 8 | 5 | 10.74 [5.71; 17.04] | 95.5% | <0.001 | 72.0 [52.5; 88.2] | 93.1% | 0.627 |
|  | Asia | 4 | 11 | 1 149 | 2 | 2 | 0.23 [0.00; 0.67] | 0% |  | 47.0 [0.0; 100] | 43.3% |  |
|  | Latin America | 66 | 94 | 571 | 2 | 2 | 12.05 [1.54; 31.39] | 96.6% |  | 73.3 [20.3; 100] | 96.6% |  |
|  | Oceania | 47 | 120 | 487 | 2 | 1 | 12.77 [0.06; 40.12] | 97.5% |  | 43.3 [4.8; 87.5] | 96.4% |  |
| **Country income level*** | |  |  |  |  |  |  |  |  |  |  |  |
|  | Low income | 156 | 277 | 3 592 | 5 | 5 | 3.79 [0.82; 8.70] | 97.2% | 0.018 | 50.1 [34.4; 65.9] | 79.2% | 0.086 |
|  | Middle income | 240 | 376 | 1 827 | 9 | 5 | 13.04 [6.89; 20.72] | 94.7% |  | 77.9 [50.9; 97.2] | 95.4% |  |
| **Setting*** | |  |  |  |  |  |  |  |  |  |  |  |
|  | Rural | 123 | 167 | 2 044 | 5 | 3 | 9.76 [1.29; 24.33] | 98.5% | 0.948 | 76.5 [40.8; 99.2] | 94.1% | 0.549 |
|  | Urban | 258 | 413 | 3 013 | 8 | 8 | 9.40 [5.51; 14.17] | 92.6% |  | 66.4 [49.5; 81.6] | 87.3% |  |
| **Study year*** | |  |  |  |  |  |  |  |  |  |  |  |
|  | 1998 - 2011 | 250 | 448 | 4 082 | 8 | 7 | 7.70 [3.41; 13.45] | 96.9% | 0.570 | 61.1 [36.6; 83.2] | 95.8% | 0.418 |
|  | 2012 - 2022 | 146 | 205 | 1 337 | 6 | 5 | 10.89 [2.75; 23.27] | 97.1% |  | 76.1 [57.1; 91.7] | 78.5% |  |
| **Number of symptoms assessed*** | | |  |  |  |  |  |  |  |  |  |  |
|  | Between 1 and 4 | 114 | 225 | 1 342 | 5 | 5 | 7.09 [2.45; 13.79] | 93.8% | 0.416 | 59.4 [19.9; 93.9] | 95.7% | 0.442 |
|  | Five and more | 261 | 382 | 3 716 | 8 | 5 | 10.97 [4.40; 19.91] | 98.0% |  | 73.6 [54.7; 89.1] | 91.3% |  |
| **Key population**** | |  |  |  |  |  |  |  |  |  |  |  |
|  | Pregnant women | 129 | 253 | 1 268 | 7 | 3 | 10.89 [5.31; 18.06] | 92.0% | .. | 63.6 [39.1; 85.2] | 92.2% | .. |
|  | Female sex workers | 37 | 138 | 546 | 2 | 2 | 7.02 [4.16; 10.52] | 41.3% | .. | 26.7 [19.6; 34.5] | 0% | .. |
|  | Adolescents | 13 | 28 | 706 | 2 | 2 | 2.05 [0.33; 4.98] | 73.7% | .. | 46.4 [27.6; 65.7] | 0% | .. |
|  | Women with HIV | 47 | 64 | 382 | 2 | 1 | 9.02 [0.18; 27.51] | 95.4% | .. | 75.1 [62.5; 86.1] | 0% | .. |
|  | Infertile | 1 | 1 | 137 | 1 | 1 | 0.73 [0.04; 4.60] | .. | .. | 100 [5.5; 89.2] | .. | .. |

* Excludes populations with an increased risk of STI (FSW, women with HIV, and women attending an STI clinic)
** "Pregnant women" and "Women with HIV" are not mutually exclusive
