# Supplementary material for: Asymptomatic infections with Chlamydia trachomatis, Neisseria gonorrhoeae, and Trichomonas vaginalis among women in low- and middle-income countries: A systematic review and meta-analysis
Source: PLOS Glob Public Health. 2024 May 23;4(5):e0003226. doi: 10.1371/journal.pgph.0003226 (PMC11115196; doi:10.1371/journal.pgph.0003226)
Supplement: S10 Table — Meta-regression estimates and 95% CI for the proportion and the prevalence of asymptomatic CT (A), NG (B), and TV (C). (DOCX) [file pgph.0003226.s013.docx]

**S10 Table: Meta-regression estimates and 95% CI for the proportion and the prevalence of asymptomatic CT (A), NG (B), and TV (C)**

**A**

|  | **Proportion of asymptomatic CT** | |  | **Prevalence of asymptomatic CT** | |
| --- | --- | --- | --- | --- | --- |
|  | **Estimate** | **95% CI** |  | **Estimate** | **95% CI** |
| **Continent** |  |  |  |  |  |
| Africa | Ref | - |  | - | - |
| Asia | 0.022 | (-0.022; 0.264) |  | 0.014 | (-0.064; 0.091) |
| Latin America | -0.328 * | (-0.639; -0.016) |  | -0.102 * | (-0.205; 0.001) |
| Oceania | 0.210 | (-0.307; 0.728) |  | 0.216 * | (0.030; 0.403) |
| **Country income level** |  |  |  |  |  |
| Low income | Ref | - |  | - | - |
| Middle income | 0.246 * | (0.044; 0.448) |  | 0.115 *** | (0.049; 0.181) |
| **Setting** |  |  |  |  |  |
| Rural | Ref | - |  | - | - |
| Urban | -0.156 | (-0.405; 0.093) |  | -0.068 * | (-0.147; 0.011) |
| **Study year** |  |  |  |  |  |
| 1998 - 2011 | Ref | - |  | - | - |
| 2012 - 2022 | -0.206 * | (-0.404; -0.008) |  | -0.017 | (-0.082; 0.049) |
| **Number of symptoms assessed** |  |  |  |  |  |
| <5 | Ref | - |  | - | - |
| 5 and more | -0.023 | (-0.228; 0.183) |  | -0.102 ** | (-0.170; -0.035) |

**B**

|  | **Proportion of asymptomatic NG** | |  | **Prevalence of asymptomatic NG** | |
| --- | --- | --- | --- | --- | --- |
|  | **Estimate** | **95% CI** |  | **Estimate** | **95% CI** |
| **Continent** |  |  |  |  |  |
| Africa | Ref | - |  | - | - |
| Asia | -0.468 ** | (-0.750; -0.185) |  | -0.155 | (-0.312; 0.002) |
| Latin America | -0.469 * | (-0.885; -0.052) |  | -0.124 | (-0.346; 0.099) |
| Oceania | 0.045 | (-0.411; 0.502) |  | 0.107 | (-0.194; 0.408) |
| **Country income level** |  |  |  |  |  |
| Low income | Ref | - |  | - | - |
| Middle income | 0.254 * | (0.051; 0.458) |  | 0.202 ** | (0.075; 0.328) |
| **Setting** |  |  |  |  |  |
| Rural | Ref | - |  | - | - |
| Urban | -0.187 | (-0.394; 0.019) |  | -0.145 * | (-0.280; -0.010) |
| **Study year** |  |  |  |  |  |
| 1998 - 2011 | Ref | - |  | - | - |
| 2012 - 2022 | -0.327 ** | (-0.543; -0.111) |  | -0.064 | (-0.193; 0.065) |
| **Number of symptoms assessed** |  |  |  |  |  |
| <5 | Ref | - |  | - | - |
| 5 and more | 0.302 ** | (0.075; 0.530) |  | -0.076 | (-0.204; 0.052) |

**C**

|  | **Proportion of asymptomatic TV** | |  | **Prevalence of asymptomatic TV** | |
| --- | --- | --- | --- | --- | --- |
|  | **Estimate** | **95% CI** |  | **Estimate** | **95% CI** |
| **Continent** |  |  |  |  |  |
| Africa | Ref | - |  | - | - |
| Asia | -0.361 | (-0.790; 0.067) |  | -0.275 *** | (-0.404; -0.145) |
| Latin America | -0.148 | (-0.583; 0.287) |  | -0.085 | (-0.242; 0.072) |
| Oceania | 0.104 | (-0.588; 0.797) |  | 0.261 | (-0.002; 0.525) |
| **Country income level** |  |  |  |  |  |
| Low income | Ref | - |  | - | - |
| Middle income | 0.183 | (-0.158; 0.524) |  | 0.120 | (-0.001; 0.241) |
| **Setting** |  |  |  |  |  |
| Rural | Ref | - |  | - | - |
| Urban | -0.233 | (-0.591; 0.125) |  | -0.101 | (-0.230; 0.028) |
| **Study year** |  |  |  |  |  |
| 1998 - 2011 | Ref | - |  | - | - |
| 2012 - 2022 | -0.046 | (-0.363; 0.271) |  | -0.049 | (-0.159; 0.061) |
| **Number of symptoms assessed** |  |  |  |  |  |
| <5 | Ref | - |  | - | - |
| 5 and more | -0.086 | (-0.409; 0.237) |  | -0.059 | (-0.174; 0.055) |
