# Supplementary material for: Asymptomatic infections with Chlamydia trachomatis, Neisseria gonorrhoeae, and Trichomonas vaginalis among women in low- and middle-income countries: A systematic review and meta-analysis
Source: PLOS Glob Public Health. 2024 May 23;4(5):e0003226. doi: 10.1371/journal.pgph.0003226 (PMC11115196; doi:10.1371/journal.pgph.0003226)
Supplement: S2 Fig — (DOCX) [file pgph.0003226.s015.docx]

**S2 Fig: Funnel plot and Egger’s test for the proportion of asymptomatic NG**

**
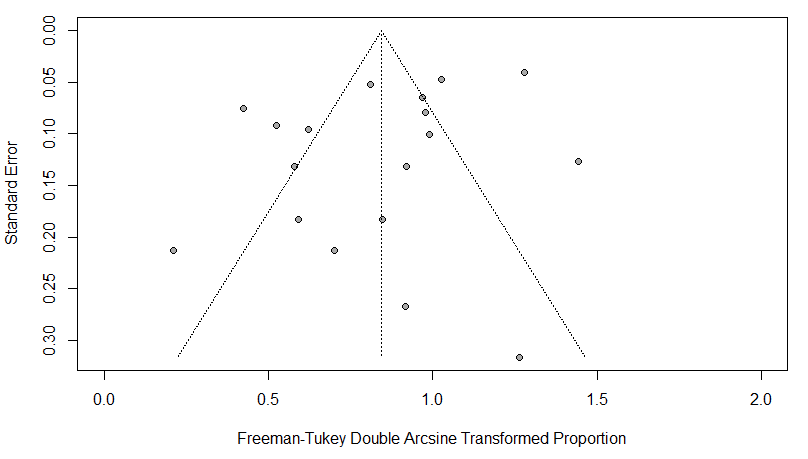
**

Egger’s test: p = 0.118
Eggers' test does not indicate the presence of funnel plot asymmetry.
